# The Cxcl14 chemokine defines pioneer axon guidance and early circuit assembly in the inner ear

**DOI:** 10.1083/jcb.202604185

**Published:** 2026-11-11

**Authors:** Mireia Rumbo, Aitor Bañón, Alex V. Nechiporuk, Berta Alsina

**Affiliations:** 1 Department of Medicine and Life Sciences, Universitat Pompeu Fabra-Parc de Recerca Biomèdica de Barcelona, Barcelona, Spain; 2 Department of Cell, Developmental & Cancer Biology, Oregon Health & Science University, Portland, OR, USA

## Abstract

The nervous system wiring requires the precise coordination of axon guidance, neuronal migration, and target cell recognition. Here, we show that inner ear circuit formation relies on pioneer cells extending an axonal scaffold that selectively targets early-born hair cells. High spatiotemporal imaging of pioneer axons reveals how they navigate through the cranial environment, establishing dynamic cell–cell contacts with other axons to finally stabilize in target cells. These pioneer axons are required not only for follower axon growth but also for coordinated migration of follower neurons, revealing a cellular hierarchy underlying circuit assembly. We identify the chemokine Cxcl14 as a novel instructive guidance cue regulating pioneer axon extension, turning, and fasciculation at discrete cellular decision points. Loss of Cxcl14 disrupts axonal navigation, compromises synaptic organization in hair cells, and impairs mechanosensory-based behavior. Together, our findings establish a new chemokine-based mechanism linking pioneer axon guidance to early sensory circuit assembly necessary for building mechanosensory networks.

## Teaser

Rumbo et al. find pioneer axons of the inner ear creating a scaffolding that guides follower neurons and selectively targets early-born inner ear hair cells. Driven by the chemokine Cxcl14, this cellular hierarchy ensures proper axonal navigation, synaptic organization, and functional mechanosensory network assembly.

## Introduction

The establishment of precise neural circuits during embryogenesis requires a coordinated sequence of neuronal migration, axon extension, and navigation through complex molecular environments, combined with selective target recognition to ensure accurate signal transmission. A highly conserved mechanism for circuit development is the initial extension of pioneer axons that organize a scaffold circuit that is used for migration of later-born neurons or growth of follower axons (Hidalgo and Brand, 1997; Supèr et al., 1998).

The inner ear is a key sensory organ of our head responsible for detecting and transmitting acoustic, linear, and rotational acceleration information to the brain. This is achieved through the establishment of a mechanosensory circuit connecting hair cells (HCs) with bipolar neurons of the statoacoustic ganglion (SAG). HCs are strategically arranged in separated sensory domains within the otic epithelium to detect sensory information across the three orthogonal head axes (Fritzsch et al., 2002; Higuchi et al., 2019). As SAG neurons are placed outside the otic epithelium, axons must navigate within the cranial mesenchyme and find their target HCs. Several works have addressed the molecular mechanisms regulating SAG axonal guidance toward HCs. The generation of ectopic HCs in the inner ear epithelium of mouse embryos resulted in rerouting of SAG axons and targeting of the ectopic HCs, suggesting the secretion of a putative attractive molecule by HCs (Zheng and Gao, 2000). Although BDNF and NT3 neurotrophins, expressed in all HCs, were postulated to be the attractant signaling molecules, it was difficult to decipher whether they exerted trophic or tropic roles on SAG axons (Tessarollo et al., 2004). Conversely, in chick otic explants, SAG axons reached sensory regions before HC differentiation (Hemond and Morest, 1992), arguing for non-HC–derived guidance cues. Similarly, in mouse mutants lacking BDNF and HC differentiation, SAG axons still reached the sensory domains, suggesting that other earlier-expressed molecules in the sensory region could mediate the early guidance of SAG axons (Fritzsch et al., 2005). Taking these results into consideration, the guidance mechanisms by which SAG axons reach early-born HCs located in distinct regions are poorly understood. In addition, whether the establishment of this circuitry relies on pioneer axons remains to be explored.

Recently, we identified a population of non-otic pioneer SAG neurons (thereafter, pioneers) that are necessary for the migration and coalescence of otic neuroblasts into distinct lobes (Bañón and Alsina, 2023). While pioneers of cranial ganglia have received less attention, studies on the posterior lateral line (LL), a mechanosensory system that senses water flow through HCs organized in neuromasts and develops in close contact with the otic vesicle (OV), demonstrated that pioneer axons targeted the migrating primordium, guiding follower axons (Ghysen and Dambly-Chaudière, 2004). These posterior LL (pLL) pioneers were characterized by a specific transcriptomic signature including the expression of *ret-gfra1* receptors to mediate axonal growth and dynamics (Sato and Takeda, 2013; Tuttle et al., 2019; Woodruff et al., 2025). However, whether this transcriptional signature is conserved in other parts of the developing nervous system, including the inner ear, is not known.

Historically, chemokines have been mainly described as directing immune cell migration (Hughes and Nibbs, 2018), but recent studies have explored other roles in cell differentiation, survival, synaptic activity, and axon guidance (Borrell and Marín, 2006; Li et al., 2005; Rostène et al., 2011). The Cxcl12-Cxcr4 signaling axis, for instance, is present in organisms before the appearance of an immune system and is a regulator of neural development (Li and Ransohoff, 2008). Cxcl14 is one of the closest homologs of Cxcl12, implicated mainly in angiogenesis, tumor growth, and migration of immune cells in adults (DeVries et al., 2006). However, its functions remain comparatively unexplored compared with other chemokines, largely because it does not seem to interact with the classical CXC-type receptors and its cognate receptor has not yet been identified (Tanegashima et al., 2013; Collins et al., 2017; Al Hamwi et al., 2024). In the mouse hippocampus, CXCL14 was described to regulate the differentiation of rare microglia (Schmid et al., 2009; Li et al., 2019). Moreover, CXCL14 has been implicated in the regulation of GABAergic synaptic transmission and differentiation of cortical interneurons, modulating neuronal activity and circuit maturation (Banisadr et al., 2011; Iannone et al., 2024). Intriguingly, Cxcl14 shows a highly conserved expression pattern in HCs of the inner ear in zebrafish, *Xenopus*, mouse, and human (Park et al., 2009; Gordon et al., 2011; Wang et al., 2024), and it was also found to be enriched in regenerating HCs after damage in chick and mouse, assessed by scRNA-seq (Janesick et al., 2022; Luca et al., 2025). Its evolutionarily conserved expression in HCs and its role in synaptic activity and circuit maturation suggest a putative role in inner ear circuit formation.

In this work, we reveal that SAG pioneers share some transcriptional markers with anterior LL (aLL) progenitors, suggesting a common SAG-aLL pioneer pool involved in the development of both cranial ganglia. We also present, for the first time, high-spatiotemporal-resolution imaging of SAG pioneer axon dynamics, revealing their initial contact with HCs and demonstrating their requirement for the subsequent migration of otic neuroblasts. Overall, these results highlight a fundamental role of pioneer axons in the establishment of the scaffold for the organization of the future mechanosensory circuit. Finally, we identify the Cxcl14 chemokine as a central cue regulating pioneer axon guidance. In *cxcl14*-deficient embryos, SAG pioneer axons exhibited pathfinding errors, reduced fasciculation, and turning errors. At later developmental stages, branching complexity at innervation sites was diminished, accompanied by incorrect HC targeting, a reduction in HC activity, and synaptic disorganization. Moreover, *cxcl14*-deficient embryos also displayed strong phenotypes in the LL and SAG central fascicles projecting to the hindbrain. Disruption of the circuitry resulted in a significantly reduced startle response, indicating a functional impairment of the vestibuloacoustic system. Together, our findings reveal the existence and relevance of pioneer axonal circuit assembly in the inner ear, and we identify Cxcl14 as the first molecule regulating both peripheral and central projections of SAG pioneer axons.

## Results

### Pioneer SAG neurons share a transcriptional profile with aLL progenitors

**Figure 1.**
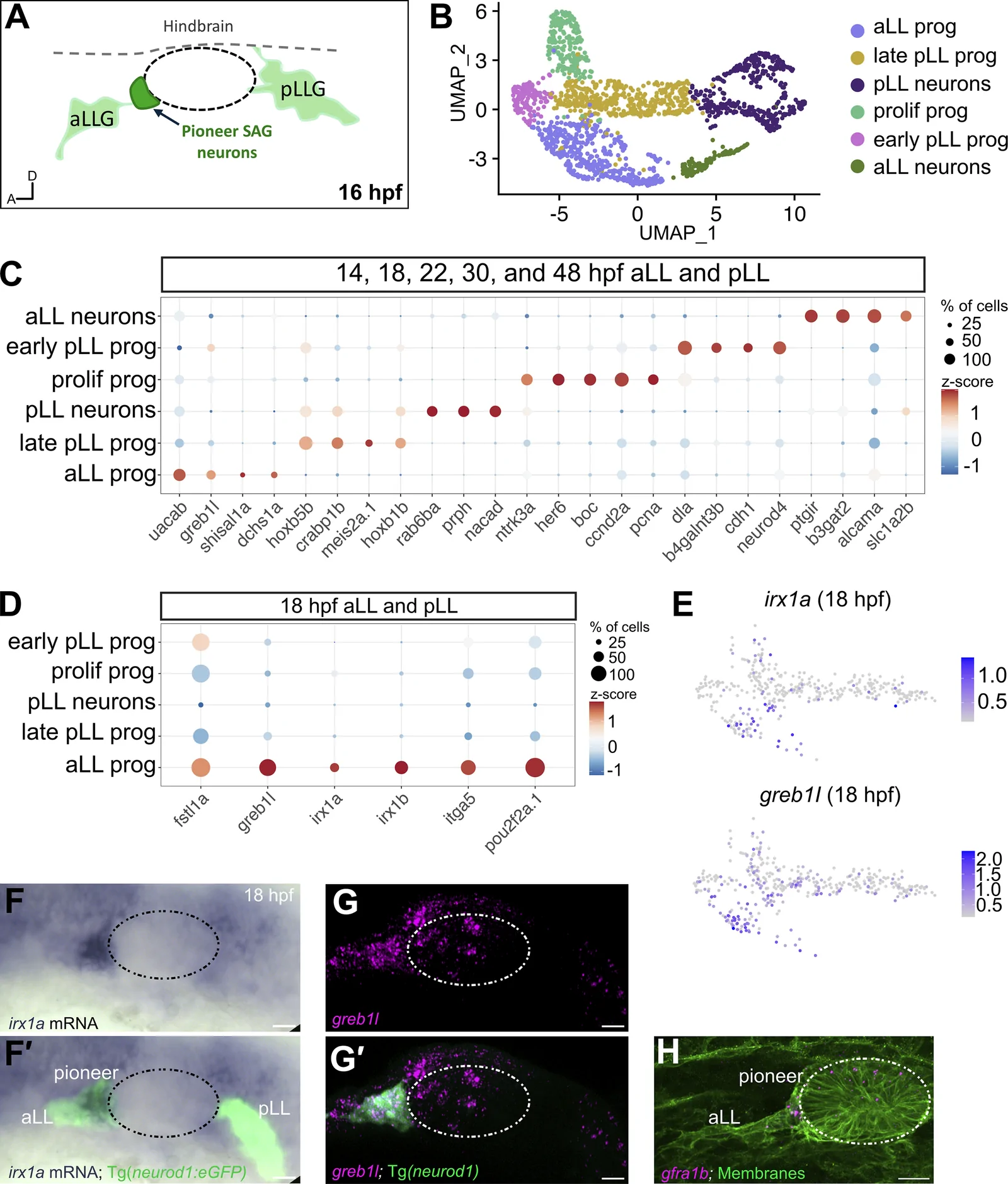
**SAG pioneers share a transcriptional profile with aLL progenitors. (A)** Schematic representation of pioneer SAG neurons (dark green), aLL, and pLL ganglia (light green) at 16 hpf. **(B)** UMAP of lateral line, TgBAC(*neurod1:eGFP*)^nl1^-positive cells from 14, 18, 22, and 48 hpf clustered as aLL progenitors, late pLL progenitors, pLL neurons, proliferating progenitors, early pLL progenitors, and aLL neurons. **(C)** Dot plot showing expression of enriched markers in each subcluster. **(D)** Dot plot showing expression of known SAG markers in the lateral line in the 18 hpf data set. **(E)** Feature plots showing expression of *irx1a* at 18 hpf. **(F and F′)***irx1a* expression in pioneers (dark blue) with sensory neurons visualized by TgBAC(*neurod1:eGFP*)^nl1^ (green) at 18 hpf. **(G and G′)** HCR labelling of *greb1l *(magenta) with sensory neurons visualized by TgBAC(*neurod1:eGFP*)^nl1^ (green) at 18 hpf. **(H)** FISH labelling of *gfra1b* (magenta), a pioneer marker, at 18 hpf. Membranes are labelled by the *cldnB:memgfp* transgene (green). aLLG, anterior lateral line ganglion; pLLG, posterior lateral line ganglion. Dotted circle represents OV.

We recently identified a group of non-otic pioneer cells, positive for *neurod1*, positioned anterior to the otic primordium that orchestrate the migration and coalescence of later inner ear delaminating neuroblasts (Bañón and Alsina, 2023) (Fig. 1 A). However, the transcriptomic identity of these pioneer cells had not been addressed. As SAG pioneers develop next to the aLL ganglion, we wondered whether the SAG pioneers and aLL progenitors share their lineage and transcriptomic profile. To assess whether this was the case, we analyzed scRNA-seq datasets from the LL published in Woodruff et al. (2025) to identify aLL neurons and their progenitors from embryos at distinct stages. We subclustered previously annotated LL cells and used differential expression analysis and previously defined markers to identify aLL and pLL neurons as well as their progenitor populations (Fig. 1, B and C). For example, pLL lineage displays high levels of *hoxb5b*, whereas aLL progenitors and aLL neurons express high levels of *alcama* compared with the pLL (Fig. 1 C) (Woodruff et al., 2025). As SAG pioneers are evident at 18 hpf, we asked whether any known SAG genes are expressed in the developing aLL at this stage, potentially marking SAG pioneers. Differential expression analysis identified several aLL transcripts, including *pou2f2a.1*, *irx1a* and *irx1b*, *greb1l*, *itga5*, and *fstl1a* (p adjusted value <0.001; Fig. 1, D and E). Whereas *both irx1a* and *greb1*l were expressed in aLL progenitors, only *irx1a* exclusively labelled the SAG pioneers (Fig. 1, F–G′). Moreover, we found that the pLL pioneer marker *gfra1b* (Woodruff et al., 2025) is also expressed in SAG pioneers (Fig. 1 H). Altogether, we concluded that SAG pioneers share markers with other sensory pioneers, suggesting that pioneer identity represents a conserved cellular program deployed across distinct mechanosensory systems.

### Pioneer neurons extend pioneer axons that target first-born HCs and serve as scaffolds for the migration of otic neuroblasts

**Figure 2.**
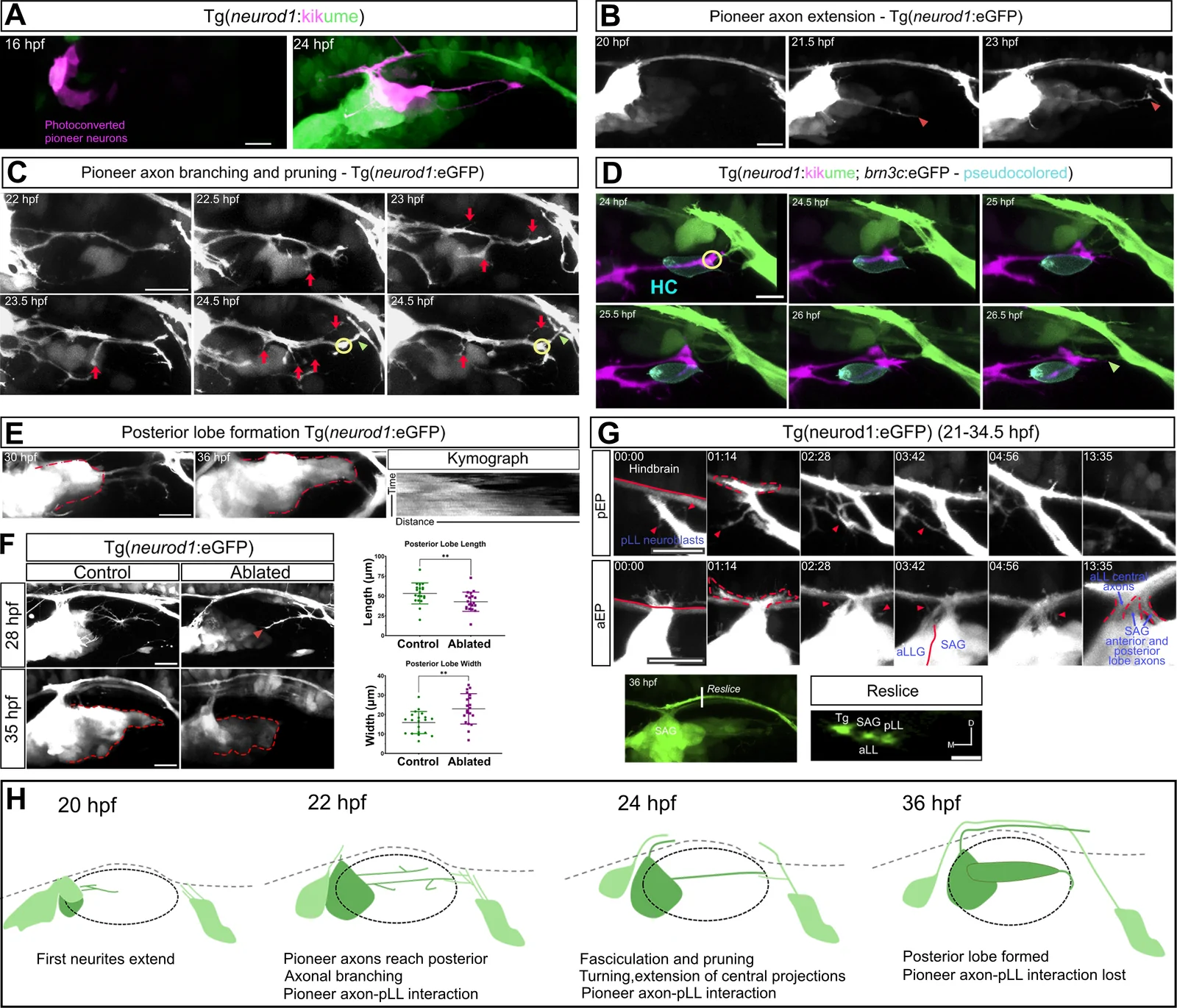
**SAG pioneer axons target newborn HC and serve as scaffolds for neuroblast migration. Central projections grow simultaneously in a stereotyped manner. (A)** Photoconversion from green to magenta of the SAG pioneers using the Tg(*neurod1:kikume*) line. Visualization of the photoconverted neurons at 24 hpf, in which SAG pioneer neurons (magenta) extend the first pioneer axons of the SAG, targeting the posterior region of the OV (white arrow labelling the growth cone). **(B)** Representative stills from a confocal time-lapse of the SAG pioneer axon growth to the posterior region of the OV from 20 to ≈24 hpf. Sensory neurons and axons are visualized by TgBAC(*neurod1:*eGFP)^nl1^. Scale bar = 20 µm. Red arrowheads mark the growth cone of the axon. **(C)** Representative stills of a confocal time-lapse showing branching and pruning of SAG pioneer axons (red arrows) from 22 to 24.5 hpf. The yellow circle indicates the thickening of the axon when the posterior sensory region is reached. The green arrowhead indicates the contact between SAG pioneer axons and pLL axons. Scale bar = 10 µm. **(D)** Photoconverted SAG pioneer axon (magenta) targets and contacts the first newborn HC (pseudocolored in cyan). A thickening in the axon is observed at the base of the HC (yellow circle). pLL (green) axons that contact SAG pioneer axons are depicted by a green arrowhead. Scale bar = 10 µm. **(E)** Stills showing the crawling of neuroblasts along the SAG pioneer axons to form the posterior lobe (marked with a red dotted line). Kymograph representing how the axon gets populated by neuroblasts through time. **(F)** Confocal images from control and pioneer neuron-ablated embryos at 28 and 35 hpf. Arrow indicates pLL axons entering otic territory. Red dotted line indicates posterior lobe shape. Quantifications for posterior lobe length and width are shown in the graphs on the right. *N* = 20 controls and 20 ablated. Lobe length: control = 53.18 ± 13.2 and ablated = 42.72 ± 12.2, **P value = 0.0039. Lobe width: control = 15.85 ± 5.7 and ablated = 22.98 ± 7.8, **P value = 0.0026. Values are given as mean ± SD. Scale bar = 20 µm. **(G)** Still confocal images from a time series showing the growth and entrance to the hindbrain of central projections from pLL and aLL-SAG, respectively, in the hindbrain entry points between 22 and 35.5 hpf. Arrowheads = temporal branches. Dashed lines are used to mark central axons when they turn. Scale bar = 20 µm. Confocal image of a 36 hpf SAG from a lateral view and the orthogonal view (reslice) of the fasciculated central projections of Tg, aLL, SAG, and pLL. **(H)** Schematic representation of the dynamics of growth of the SAG and the central projections from aLL and pLL from 20 to 36 hpf. pEP, posterior entry points; aEP, anterior entry points.

**Figure S1.**
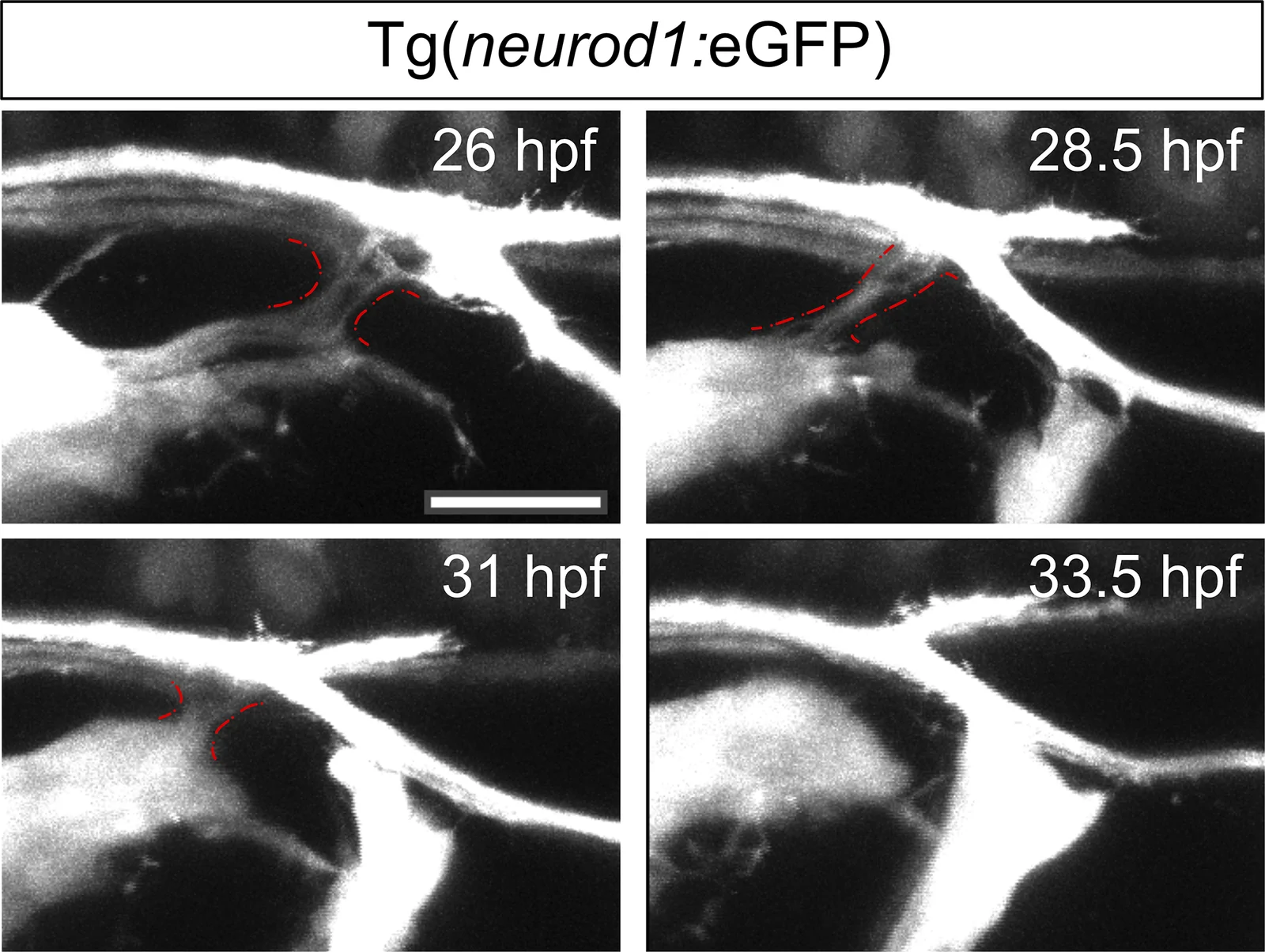
**Disassembly of SAG axons and pLL contacts.** Representative confocal stills of a time series from 26 to 33.5 hpf of TgBAC(*neurod1:eGFP*)^nl1^. Axonal contacts are marked with red dotted lines. Scale bars = 20 µm.

We then explored whether the SAG pioneers establish the first scaffolding axonal circuit. For this, we labeled the pioneer cells via photoconversion using the transgenic (Tg)(*neurod1:kikume)* line. Indeed, the first axons extending posteriorly were exclusively marked by a photoconverted fluorophore, confirming their pioneer identity (Fig. 2 A, white arrow). To better understand how pioneer axons extend to HCs, we performed confocal time-lapse acquisitions at high spatiotemporal resolution. We focused on the extension of the pioneer SAG axons to the posterior region of the OV. Initially, one or two neurites extended posteriorly (arrowhead marks the axon growth cone; Fig. 2 B and Video 1). During this period, the extending pioneer axon also produced many shorter perpendicular branches that finally retracted (Fig. 2 C, red arrows; Video 2). We confirmed that these pioneer axons contacted the first-born HC by photoconverting the SAG pioneers and simultaneously labelling HCs with the Tg(*Brn3C:GFP*)^*s356t*^ reporter (Fig. 2 D). A thickening of the axon near the posterior newborn HC was visible, indicative of the first axon-HC contact (Fig. 2, C and D, yellow circles). Surprisingly, the SAG pioneer axon did not stop at that contact but extended posteriorly, passing past the inner ear and establishing thinner contacts with the hindbrain and axonal branches from the pLL central projection (Fig. 2, C and D, green arrowheads). The pioneer SAG axon–pLL axon interaction was finally resolved by 33.5 hpf (Fig. S1 and Fig. 6). The process of SAG pioneer axon extension, branching/refinement, retraction, and final stabilization to the HC lasted 5 h, from 20 to 25 hpf.

**Video 1.** **Time-lapse of confocal microscopy images showing the growth of pioneer axon to the posterior region of the OV from 20 to 23 hpf (stills of this video are shown in Fig. 2 B).** Red arrowheads label axon growth tips. Scale bar = 20 µm. Frames were obtained every 8 min.

**Video 2.** **Time-lapse of confocal microscopy images showing branching of pioneer axons from 22 to 24.5 hpf (stills of this video are shown in Fig. 2 C).** Red arrowheads indicate branches. Scale bar = 20 µm. Frames were obtained every 16 min.

Remarkably, starting at 24 hpf, newly delaminated neuroblasts crawled along the pioneer axons to form the posterior lobe (Fig. 2 E, posterior lobe marked with a red dotted line; Video 3). To address whether pioneer axons were required for this process, we ablated SAG pioneers at 16 hpf, which resulted in the absence of the pioneer axons at 28 hpf. In some ablated embryos, we observed a long branch of the pLL central bundle entering the otic domain (Fig. 2 F, red arrowhead). Quantification of the posterior lobe revealed a reduction in the length of the posterior lobe, while it was wider (Fig. 2 F), suggesting that newly delaminated neuroblasts could not migrate posteriorly when the pioneer axon was missing. In some extreme cases, the posterior lobe was absent (Fig. 2 F). Altogether, the data indicate that pioneers are fundamental for neuroblast migration and ultimately organizing the lobular shape of the SAG.

**Video 3.** **Time-lapse of confocal microscopy images showing the crawling of neuroblasts along the pioneer axons to form the posterior lobe of the SAG from 30 to 36 hpf (two representative stills are shown in Fig. 2 E).** Scale bar = 10 µm. Frames were obtained every 15 min and 45 s.

We also analyzed the extension of central SAG and pLL projections, since it occurs simultaneously with peripheral ones. Small central axons began to grow at 20 hpf, initially defasciculated and emitting dynamic branches (Fig. 2 G, red arrowheads). Progressively, the central pLL axons fasciculated into a bundle, while the pLL ganglion displaced posteriorly (Fig. 2 G). The SAG and LL central projections entered the hindbrain, turned, and grew in parallel to the hindbrain border and lateral to the Tg bundle. The entry points of central projections to the hindbrain are referred to as the anterior and posterior entry points (aEP and pEP, respectively, Fig. 2 G). Three separate bundles could be observed in an orthogonal slice of the sensory axons in the hindbrain: the Tg, the aLL-SAG, and the pLL bundle, from medial to lateral as described previously (Fig. 2 G) (Zecca et al., 2015).

Overall, our live imaging of SAG and LL extending axons to their target reveals new information on how the mechanosensory circuit develops: (1) SAG pioneer axons are the first to contact HC but also establish interactions with pLL central projections; (2) peripheral and central projections progressively fasciculate into a bundle; and (3) otic neuroblasts use the SAG pioneer axons to migrate and establish the posterior lobe of the SAG (Fig. 2 H).

### Cxcl14 is an early chemokine dynamically expressed in first-born HCs and hindbrain entry points

**Figure 3.**
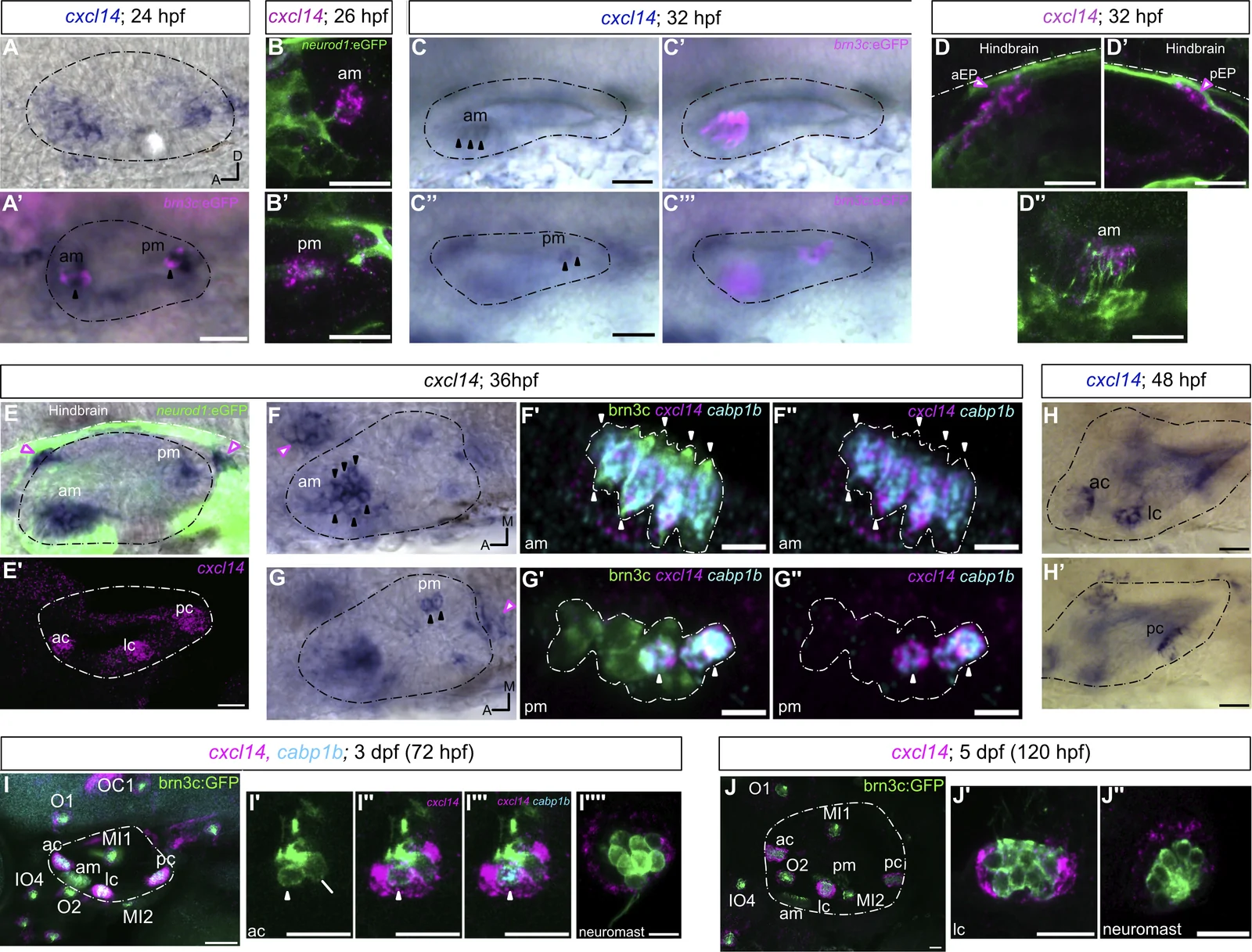
***cxcl14* is expressed in inner ear HCs and hindbrain entry points.** Chromogenic and fluorescent *in situ* hybridization (ISH) of *cxcl14* mRNA at different time points. **(A and A′)***cxcl14* is expressed in tether HCs of the am and pm at 24 hpf (black arrowheads). HCs are visualized in magenta in A′. **(B and B′)** Fluorescent ISH at 26hpf with neurons labelled in green showing the am (B) and the pm (B′) and how *cxcl14*^+^ HCs are innervated by pioneer axons. **(C–C‴)** Images at 32 hpf with the focus on the anterior region of the OV (C and C′) and the posterior region (C″ and C‴). HCs are visualized in magenta. HCs expressing *cxcl14* are marked with black arrowheads. **(D–D″)** Fluorescent ISH at 32hpf with neurons labelled in green showing expression at the anterior entry point (D, magenta and white arrowheads), posterior entry point (D, magenta and white arrowheads) and am (D″). **(E and E′)** Images at 36 hpf with neurons and axons labelled in green (E). In E′, *cxcl14* expression is observed in prospective cristae. **(F-F″)** Image with the anterior region of the OV in focus to show *cxcl14* expression in the am and anterior entry point (marked by magenta and white arrowheads). HCs expressing the chemokine are marked with black arrowheads. In F′–F″, *cxcl14* (magenta) and *cabp1b* (cyan) are co-expressed in am HCs (green, white arrowheads). **(G–G″)** Image with the posterior region of the OV in focus to show *cxcl14* expression in the pm, which is only visible in two HCs, marked with black arrowheads. Posterior hindbrain entry point is marked by magenta and white arrowheads. In G′ and G″, *cxcl14* (magenta) and *cabp1b* (cyan) are co-expressed in two HCs of the pm (white arrowheads), while more HCs (green) are already differentiated. **(H and H′)** Expression of *cxcl14 *at 48 hpf is found in the cristae. **(I–I″′′)** Expression of *cxcl14* and *cabp1b* at 72 hpf. c*xcl14* is highly expressed in cristae, and some expression can be seen in neuromasts. **(I′–I‴)** show an inset of the ac, with HCs labelled in green. White arrow in I′ indicates a HC that does not express *cxcl14 *nor *cabp1b*. **(I″)** shows expression of *cxcl14 *(magenta) in a HC (white arrowhead) and surrounding cells. **(I″′)** shows that the *cxcl14*+ HC also expresses *cabp1b* (cyan, white arrowhead). **(I″″)** shows an inset of a neuromast, in which it can be observed that *cxcl14* (magenta) is not found inside HCs (green), but in the surroundings. **(J–J″)** Expression of *cxcl14* at 5 dpf. Expression is maintained in cristae, but not inside HCs anymore (J′). Neuromasts still have mild expression surrounding HCs. Black dotted lines depict the OV silhouette. In F and G, white dotted lines depict the am and pm silhouette. ac, anterior crista; lc, lateral crista; pc, posterior crista; O2, IO4, MI1, MI2: cranial neuromasts. Neurons are labelled through TgBAC(*neurod1:eGFP*)^nl1^ and HC through Tg(*brn3c:eGFP*) ^*s356t*^. Scale bars = 20 µm. Scale bars for I and J = 50 µm.

When screening for factors that might direct pioneer SAG axons, we found that the chemokine *cxcl14* was one of the earliest molecules expressed at the first-born HCs of the inner ear. The pattern of *cxcl14* expression in the inner ear is dynamic and complex. At 24 hpf, *cxcl14* was restricted to a few cells of the prospective anterior and posterior macula domains (am and pm, respectively) of the OV (Fig. 3 A). The expression coincided with the first HCs labeled by Tg(*brn3c:eGFP)*^*s356t*^ (Fig. 3 A′, magenta and black arrowheads), known as tether cells, that are already innervated by axons at 26 hpf (Fig. 3, B and B′). By 32 hpf, *cxcl14* was expressed in three/four HCs of the am and two HCs of the pm (Fig. 3, C–C‴, black arrowheads). Interestingly, *cxcl14* also appeared in two spots adjacent to the OV and to the hindbrain (Fig. 3, D, D″, and E, magenta and white arrowheads). Analysis of *cxcl14* expression combined with the visualization of axons by the TgBAC*(neurod1:eGFP)*^*nl1*^ revealed tha*t cxcl14* expression in these two spots corresponded with the entrance of central projections of SAG and LL to the hindbrain. We refer to these regions as entry points (Fig. 3, D and D″, magenta and white arrowheads). At 36 hpf, *cxcl14* was still expressed in the am and at the two entry points, but, strikingly, expression in the pm remained in only one or two cells (Fig. 3, E′ and E″, black arrowheads). By co-labelling *cxcl14* with *cabp1b,* a marker for extrastriolar HCs, we observed that the expression of both mRNAs colocalized in the same HCs in am and pm. At this stage, HCs negative for *cxcl14* were also negative for c*abp1b*. We also noticed labelling in prospective cristae regions. Although HCs are still not visible in cristae by Brn3c labelling at this timepoint, axons are already growing toward them.

Later, at 48 and 72 hpf, *cxcl14* was highly expressed in the cristae, as these sensory domains differentiate later than maculae (Fig. 3, F and G). By 72 hpf, expression of *cxcl14* in the maculae had disappeared, while *cabp1b* is still present as reported (Baeza-Loya et al., 2026; Shi et al., 2023). As in the maculae, expression in cristae HCs coincides with *cabp1b*-expressing HCs but can also be detected in surrounding cells (supporting or precursors). At 5 days postfertilization (dpf), *cxcl14* is absent from HCs but remains in surrounding cells (Fig. 3 J′).

We also analyzed whether *cxcl14* is expressed in neuromast HCs at 72 hpf and 5 dpf. *cxcl14* was expressed at low levels in cells surrounding cranial neuromast HCs but not in neuromast Brn3c^+^ HCs.

Hence, the expression pattern of the *cxcl14* chemokine is highly dynamic, correlating with sites and time points of SAG pioneer axonal targeting, while disappearing once axons reach the target sites. Expression of *cxcl14* in HCs is restricted to the inner ear, while at later time points, it is retained in cristae and in supporting or precursor cells of LL neuromasts.

### Depletion of *cxcl14* results in axon growth and targeting defects in SAG pioneer axons and hindbrain-projecting axons

**Figure 4.**
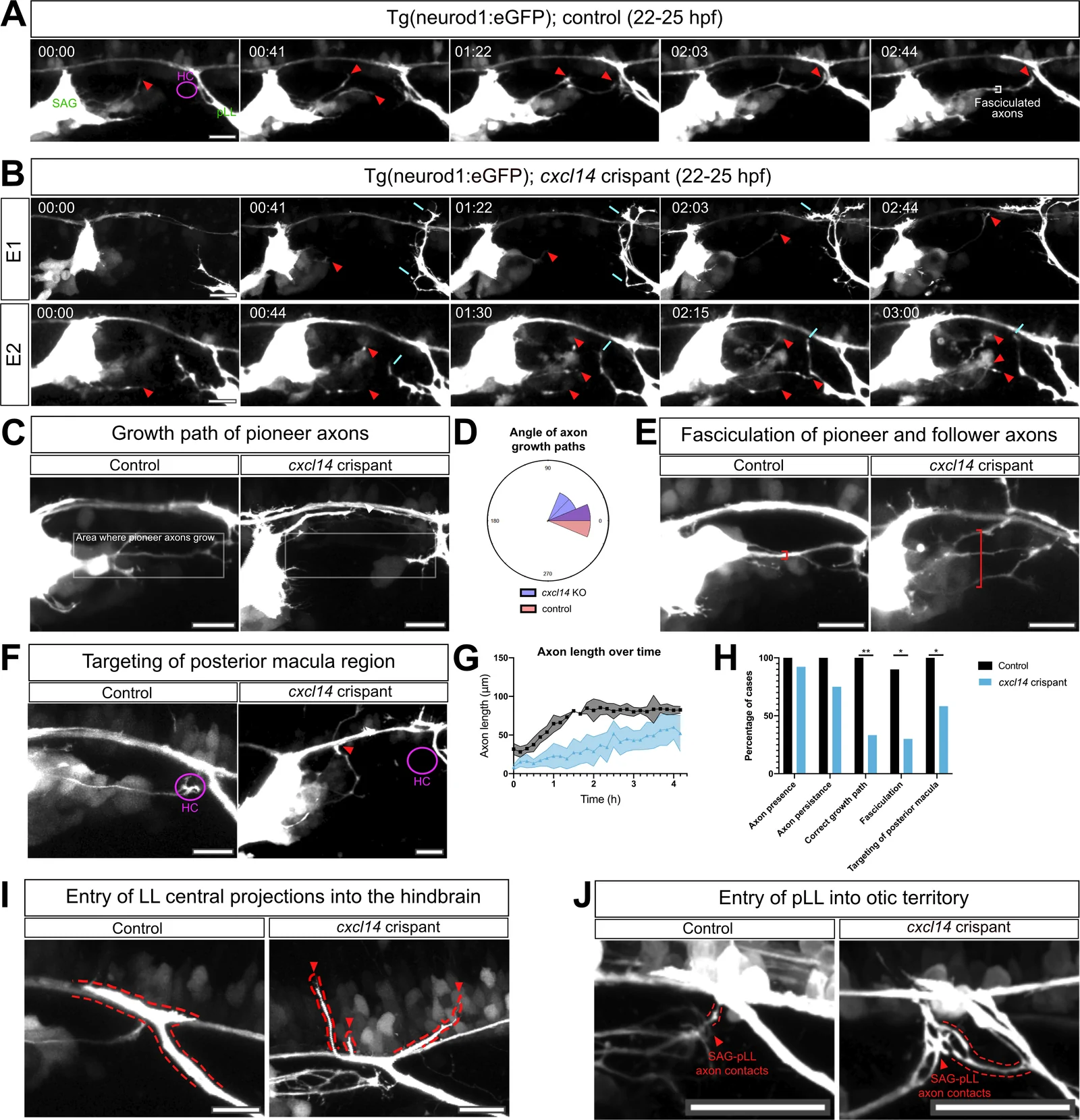
***cxcl14* depletion impairs axon growth, targeting, and fasciculation. (A and B)** Stills of a time series showing the growth dynamics of SAG pioneer axons in a control (A) and two representative examples of *cxcl14* crispants (B) from 22 to 25 hpf. Arrowheads mark the growth cone of axons. The magenta circle marks the theoretical position of the first HCs. A white square bracket indicates fasciculated axons in A. pLL axons appear defasciculated in *cxcl14 *crispants and send projections to otic territory (blue arrows). **(C)** Confocal max projections of a control and a *cxcl14* crispant. The dotted-line rectangle represents the area in which SAG pioneer axons grow in control embryos. In *cxcl14* crispant embryos, pioneer axons grow outside of this area. **(D)** Rose plot representing the angle of SAG pioneer axon growth path (control vs *cxcl14* crispant). *N* = 5 each. **(E and F)** Representative confocal max projections of a control and a *cxcl14* crispant SAG pioneer axon fasciculation (E) and targeting (F). Red square brackets in E indicate the vertical dispersion of the group of axons, from most dorsal to most medial. Magenta circles in F represent the theoretical position of newborn HC. Red arrowhead indicates the growth cone of the crispant example. **(G)** Line plot representing the SAG pioneer axon length over time of controls (grey) vs *cxcl14* crispant (light blue). *N* = 5 each. Dispersion represents SD. **(H)** Bar plot representing the percentage of cases (controls vs *cxcl14* crispant embryos) that comply with the categories: axon presence, axon persistence, correct growth path (**P value = 0.0074), fasciculation (*P value = 0.003), and targeting of pm (*P value = 0.04). *N* = 10 controls/12 *cxcl14* crispants. Fisher’s exact test was performed in all comparisons. **(I and J)** Representative confocal max projections of a control and a *cxcl14* crispant pLL axon entrance to the hindbrain (I) and to otic territory (J) (highlighted with red dotted lines). Arrowheads in I mark axons that grow dorsally in the *cxcl14* crispant. Arrowheads in J mark the contact between SAG pioneer axon and pLL axon. Scale bars = 20 µm. Tg(*neurod1:eGFP*)^nl1^ is used to label sensory neurons and axons.

The expression pattern of *cxcl14* in nascent HCs when afferent-HC connections are established, together with expression at the regions where central axons grow into the hindbrain, suggests a role of Cxcl14 in axon guidance and growth. To assess this, we performed functional analysis in *cxcl14* F_0_ CRISPR crispants (see Materials and methods). Compared with control embryos, SAG pioneer axons displayed navigation and targeting errors. A significant number of SAG axons did not follow the usual growth path (58.3%) and incorrectly targeted the posterior prosensory domain (41.7%), while we observed no errors in control cases (Fig. 4, A, F, and H). In control embryos, the growth of pioneer axons followed a horizontal and straight path towards the posterior prosensory domain (Fig.4, A and C). However, in *cxcl14* crispant embryos, growing axons explored a larger territory and grew following a tortuous path, even touching the hindbrain or growing along the central trigeminal axons (Fig. 4, B and C; and Videos 4 and 5). Thus, the angle and direction of growth in *cxcl14* crispants were wider than in controls (Fig. 4, D and E). Misrouted axons never grew towards the underlying mesenchyme. The growth of SAG pioneer axons in crispant embryos was also slower and showed more dynamic behavior, as axons tended to elongate and retract constantly, exhibiting unstable pathfinding behavior (Fig. 4 G). The exploration of more territory can also be explained by a fasciculation impediment in *cxcl14*-depleted embryos (only 30% of crispant embryos showed axon fasciculation, while 90% of controls exhibited fasciculated axons) (Fig. 4, E and H).

**Video 4.** **Time-lapse of confocal microscopy showing an example of the growth of pioneer axons and pLL in a *cxcl14* crispant embryo (E1 in Fig. 4 B) from 22 to 25 hpf.** Red arrowheads indicate SAG pioneer axons. Blue arrowheads indicate axons of the pLL entering the otic territory. Scale bar = 20 µm. Frames were obtained every 16 min.

**Video 5.** **Time-lapse of confocal microscopy showing an example of the growth of pioneer axons and pLL in a cxcl14 crispant embryo (E2 in Fig. 4 B) from 22 to 25 hpf.** Red arrowheads indicate SAG pioneer axons. Scale bar = 10 µm. Frames were obtained every 15 min.

As *cxcl14* is expressed in the hindbrain entry points, we analyzed the phenotypes of pLL axons projecting to the hindbrain when this chemokine was depleted. In control embryos, all central axons entered the hindbrain, fasciculated, and turned to position themselves in parallel and lateral to the Tg axons. However, in crispant embryos, pLL axons failed to enter the hindbrain (15,4%), and only 9% of the pLL central projections entered correctly. They exhibited growth and turning errors, growing perpendicular to the anteroposterior axis, in a dorsal direction (Fig. 4 I). Also, the contacts that were established between the SAG pioneer axons and the pLL axons happened in both control and crispants, but contacts were longer in crispants, with more axons from the pLL invading the otic (Fig. 4 J).

**Figure S2.**
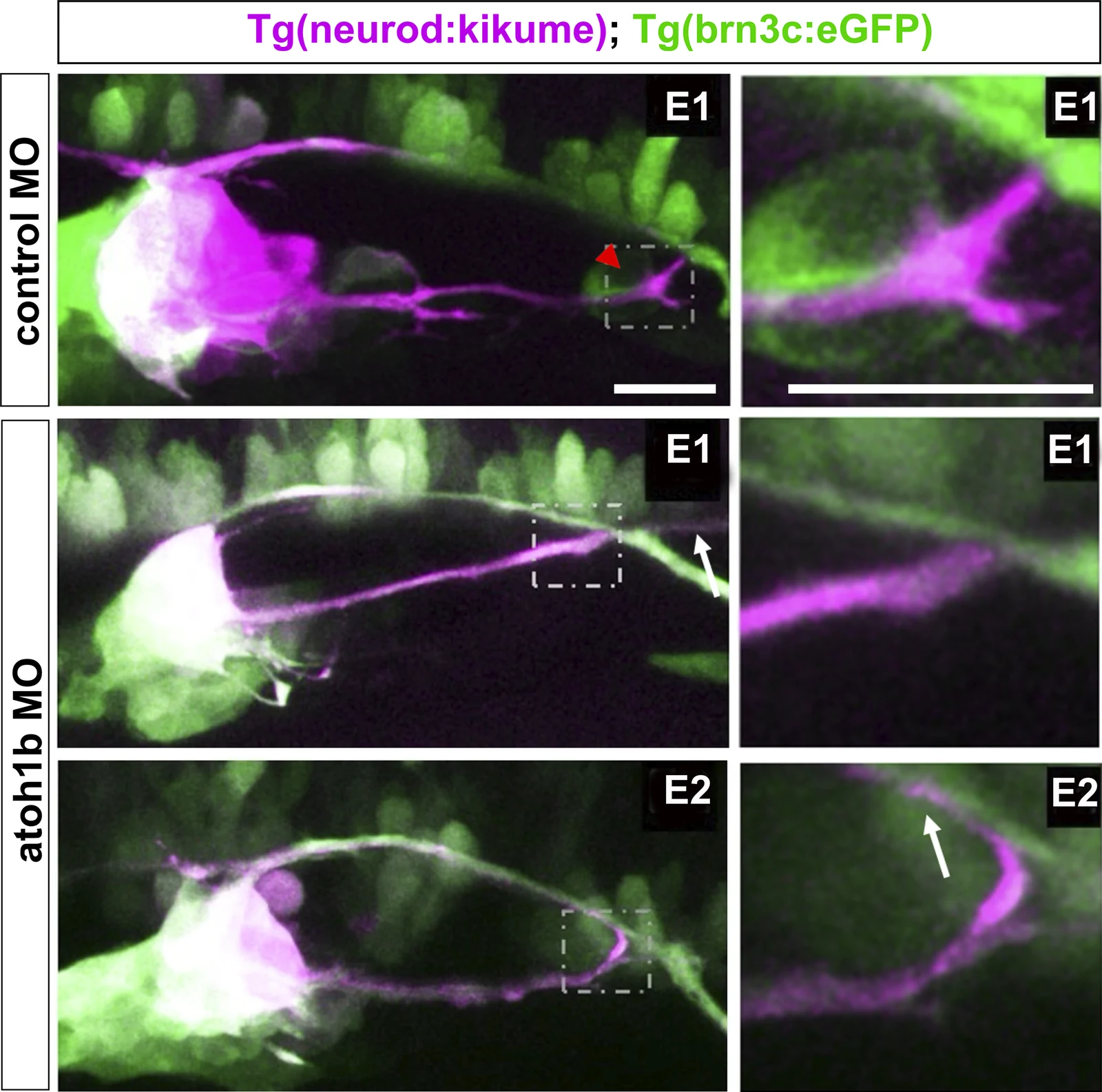
***atoh1b* depletion results in loss of HC contact by SAG pioneer axons and a consequent extended growth.** Representative confocal images of control and *atoh1b* morphants. SAG pioneers are labelled through Tg(*neurod1*:*kikume*) and photoconverted (magenta), and HCs are labelled by Tg(*brn3c*:*eGFP*) ^*s356t*^ in green. The red arrowhead depicts axon swelling. White arrows depict the extended growth of pioneer axons in atoh1b morphants. Scale bars = 20 µm.

It has been reported that *cxcl14* expression depends on *atoh1b* (Ezhkova et al., 2023). Thus, we knocked down *atoh1b* by injecting a morpholino and examined SAG pioneer axons. In *atoh1b* morphant embryos, most SAG pioneer axons extended posteriorly, and axons lost their characteristic axon swelling in the HC contacting region compared with control embryos and grew beyond the OV (Fig. S2, arrowhead in inset). As in *atoh1b* morphants, *cxcl14* is depleted from the inner ear but not from the entry points, suggesting that SAG axons could still extend posteriorly, presumably due to the non-otic posterior source of Cxcl14. As expected, in *atoh1b* morphants, pLL central projections did not present any fasciculation or turning phenotype (Fig. S2). The experiment indicates that pioneer axon initial growth is independent of HC specification, but *atoh1b* and *cxcl14* are required for proper targeting of the growth cone to HCs and preventing long-lasting connections with other growing axons. Hence, early-born sensory cells act as spatial organizers for circuit assembly, rather than passive targets of innervation.

### Ectopic expression of Cxcl14 reroutes SAG afferent axons and LL central projections

**Figure 5.**
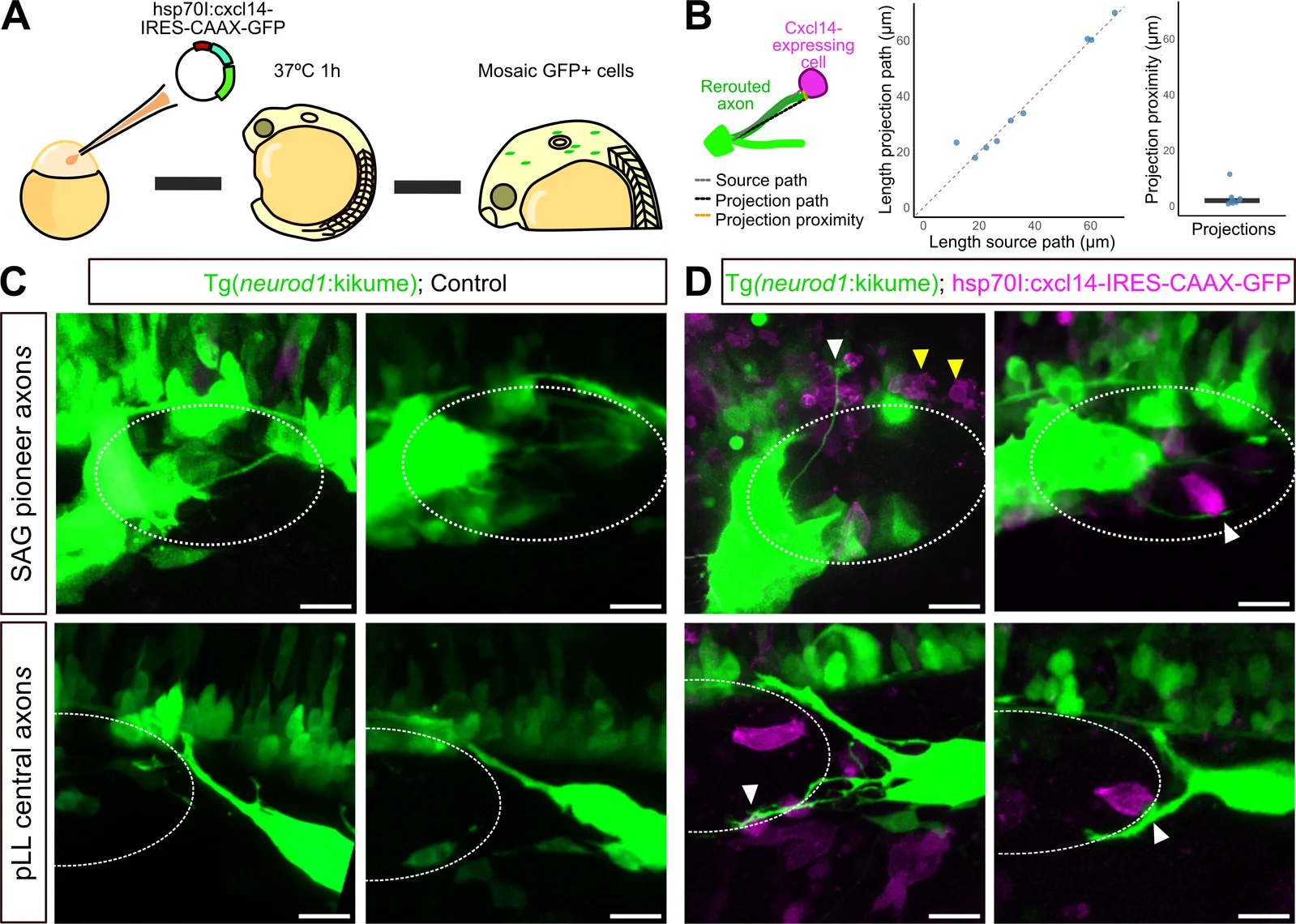
**Ectopic expression of *cxcl14* reroutes axons. (A)** Schematic representation depicting the generation of a transgenic larva that expresses *cxcl14* ectopically under the control of the Hsp70I promoter. **(B)** Dot plot showing the accuracy of eight rerouted axons in finding ectopic Cxcl14-expressing cells, based on the correlation between source path length and projection path length (µm). The second dot plot shows the distribution of projection-proximity length from the eight rerouted axons analyzed. 3.1 ± 3.2 µm (mean ± SD). **(C)** Confocal max projections of control embryos focusing on SAG axons and pLL axons. **(D)** Confocal max projections of embryos injected with hsp70I:cxcl14;IRES:CAAX-GFP plasmid and heat-shocked to ectopically express *cxcl14* (in magenta). White arrowheads indicate rerouted axons towards plasmid-expressing cells. Yellow arrowheads indicate dying cells. Scale bars = 10 µm. Neurons are visualized by photoconversion of Tg(*neurod1:kikume*) to differentiate between kikume (shown in green) and GFP from the plasmid (shown in magenta).

To test whether Cxcl14 can attract and reroute axons, we ectopically expressed Cxcl14 by injecting the *hsp70I*:*cxcl14-IRES-CAAX-GFP* plasmid, then heat-shocked embryos at 16 hpf and mounted for live imaging (Fig. 5 A). We selected embryos in which ectopic *cxcl14*-expressing cells were located near the OV (Fig. 5 C). We observed axons directed toward cells expressing Cxcl14 in 72% of the cases (*N* = 11) and, in some cases, contacting them directly (Fig. 5 C, white arrowheads). We could see that the SAG pioneer axons and the central projections of the pLL were the ones rerouting, compared with axons in controls (Fig. 5, B and C). Some axons abandoned the fascicle directed to the hindbrain and grew towards cells expressing exogenous Cxcl14 (*N* = 4, Fig. 5 C). In one case, we could even observe a SAG pioneer axon entering the hindbrain to target cells expressing Cxcl14 (Fig. 5 C). Rerouting of axons was only observed when the ectopic source of Cxcl14 was relatively nearby, possibly due to a short-range activity or other tissue constraints. To measure the accuracy of the rerouted axonal projections in finding the ectopic Cxcl14-expressing cells, we quantified the linear distance of the cell expressing exogenous Cxcl14 from the start of the rerouting (source path), together with the linear distance between the tip of the rerouted axon and its start (projection path). Then we calculated the correlation between these two distances in the eight embryos in which axons rerouted, which resulted in an almost perfect correlation (0.98). Projection paths ranged between 18.5 and 68.7 µm. Moreover, we measured the linear distance between the tip of the rerouted axon and the Cxcl14-expressing cell (projection proximity), obtaining 3.1 ± 3.2 µm (mean ± SD), which indicates that the tips of the axons were in very close proximity or in contact with the Cxcl14-expressing cells.

Based on these data, we concluded that Cxcl14 acts as an attractive signal for SAG pioneer and pLL central axons.

### Hair cell activity in cristae is compromised, and Otoferlin is mislocalized in *cxcl14* crispant embryos

**Figure 6.**
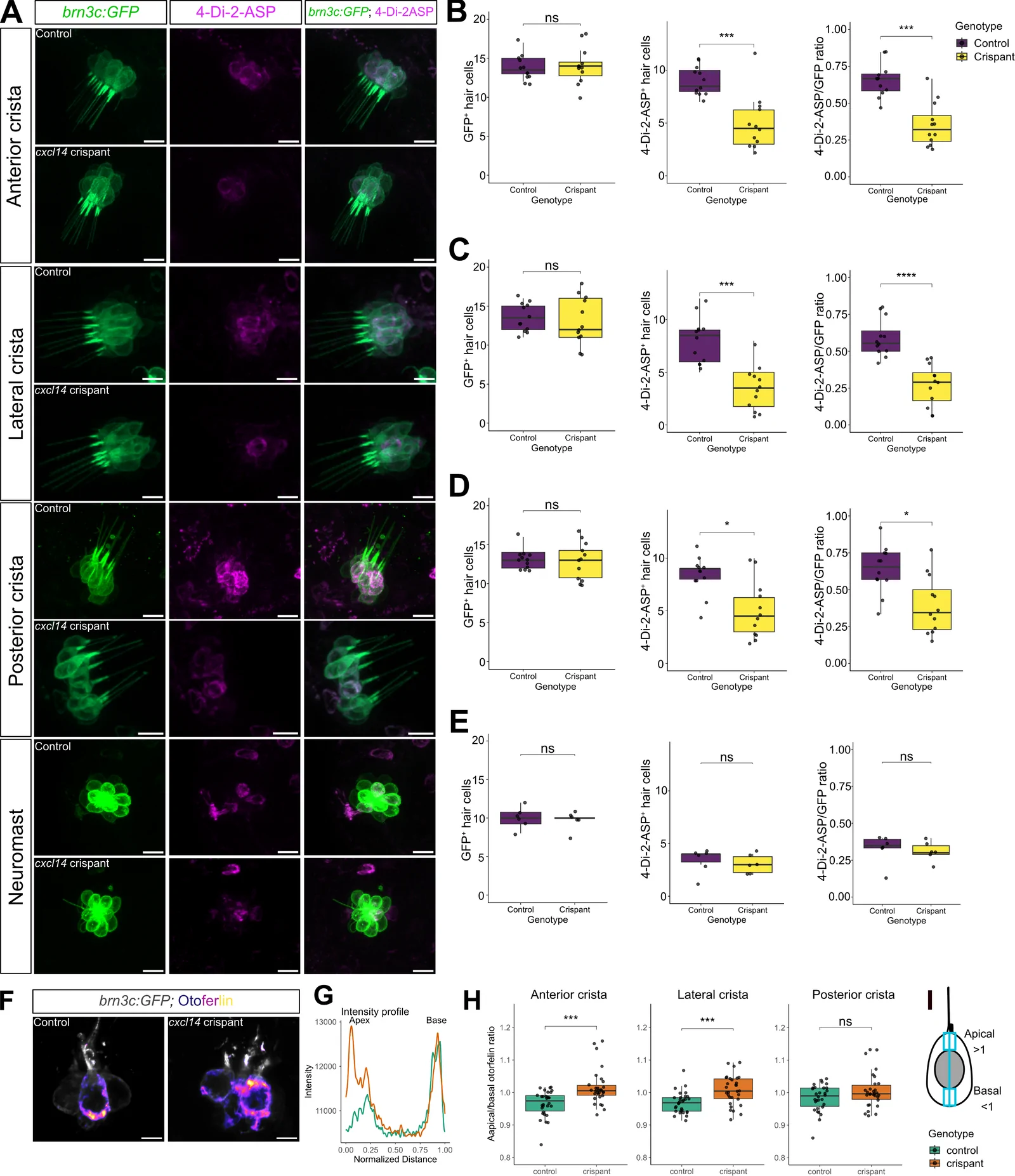
**c*xcl14* depletion has an effect on HC activity and Otoferlin distribution. (A)** Representative confocal images of anterior, lateral and posterior crista HCs labelled by Tg(*brn3c:GFP*) ^*s356t*^ (green) and 4-Di-2-ASP labelling (magenta) at 72 hpf in control and *cxcl14* crispants. Scale bar = 5 µm. **(B–E)** Boxplots showing the quantification of GFP+ HCs, 4-Di-2-ASP+ HCs, and the ratio 4-Di-2-ASP/GFP+. GFP+ HCs: ac = 13.67 ± 1.72 (controls) and 13.08 ± 3.06 (crispants), p = 0.53; lc = 13.83 ± 1.47 and 14.08 ± 2.35, p = 0.91; pc = 13.17 ± 1.19 and 12.92 ± 2.39, p = 0.52; neuromast = 10 ± 1.29 and 9.67 ± 1.24, p = 0.86. 4-Di-2-ASP+ HCs: ac = 8.08 ± 1.72 (controls) and 3.5 ± 3.06 (crispants), ***P = 0.00067; lc = 9 ± 1.37 and 5.08 ± 2.35, ***P = 0.00018; pc = 8.33 ± 1.19 and 5.08 ± 2.39, *P = 0.01; neuromas = 3.33 ± 1.1 and 3 ± 0.8, P = 0.44. Ratio: ac = 0.59 ± 0.13 (controls) and 0.27 ± 0.14 (crispants), ***P = 0.00033; lc = 0.66 ± 0.11 and 0.35 ± 0.15, ****P = 0.000067; pc = 0.64 ± 0.16 and 0.39 ± 0.19, *P = 0.01; neuromast = 0.33 ± 0.1 and 0.31 ± 0.07, P = 0.42. *N* = 12 controls/12 *cxcl14* crispants from two independent experiments. Each dot represents one embryo. **(F)** Representative z-slice confocal images of HCs labelled in grey and immunostained for Otoferlin (fire LUT). Scale bar = 5 µm. **(G)** Example of intensity profile of Otoferlin signal obtained from drawing a line from the apex to the base of HC in control (turquoise) and *cxcl14* crispant (orange). **(H)** Boxplots of the ratio between apical/basal Otoferlin signal in control (turquoise) and *cxcl14* crispants (orange). Each dot represents one cell (*n* = 30 cells from *N* = 5 embryos of each group). **(I)** Schematic representation of how the measurement of Otoferlin signal was performed in a HC. A ratio <1 = more apical expression; ratio >1 = more basal expression. Values are given as mean ± SD. Mann–Whitney test was performed in all comparisons.

The action of chemokines has been shown to be paracrine, as well as autocrine (Augsten et al., 2009), and in the central nervous system, Cxcl14 regulates calcium and GABA release (Banisadr et al., 2011; Iannone et al., 2024). Following this rationale, we assessed whether loss of *cxcl14* has any effect on HC function. HC activity was assessed with a 4-Di-2-ASP vital mitochondrial dye uptake assay, which is based on the entrance of this fluorophore through active mechanotransduction channels (Faucherre et al., 2009; Rubbini et al., 2015). While the total number of HCs (quantified by GFP labelling) did not show any significant change in *cxcl14* crispants, the ratio of active HCs/total HCs (4-Di-2-ASP+/GFP+) was significantly reduced in ac and lc (Fig. 6, A–C) and to a lesser extent in pc (Fig. 6 D). As an internal control, we also assessed the activity in HCs of the neuromasts where *cxcl14* is not expressed, and no significant differences were found in neuromast HCs (Fig. 6, A and E). The lack of inner ear HC activity in *cxcl14*-deficient embryos might indicate a defect in mechanotransducing channels or a lack of maturation of HCs.

Following our analysis of HC activity, we evaluated the localization of Otoferlin, a calcium-sensing protein that mediates synaptic vesicle release specifically in the ribbon synapses of HCs. By plotting the intensity profile of expression in a vertical line from the apical to the basal part of HCs and calculating the ratio of expression in apical/basal (Fig. 6, F–I), we found that Otoferlin was preferentially localized in the basal area of HCs in the ac and lc of control embryos, where synaptic vesicles concentrate (ratio <1, ac mean ratio = 0.96, lc mean ratio = 0.97). In *cxcl14* crispants, Otoferlin is more evenly distributed in apical and basal regions (ratio = 1.00, ac mean ratio = 1.02, lc = 1.01) (Fig. 6 H).

Thus, these results demonstrate that Cxcl14 is necessary for HC activation and synaptic organization, potentially causing subsequent failure in the transmission of mechanosensory information.

### Cxcl14 regulates the complexity of afferent axonal arbors in cristae

**Figure 7.**
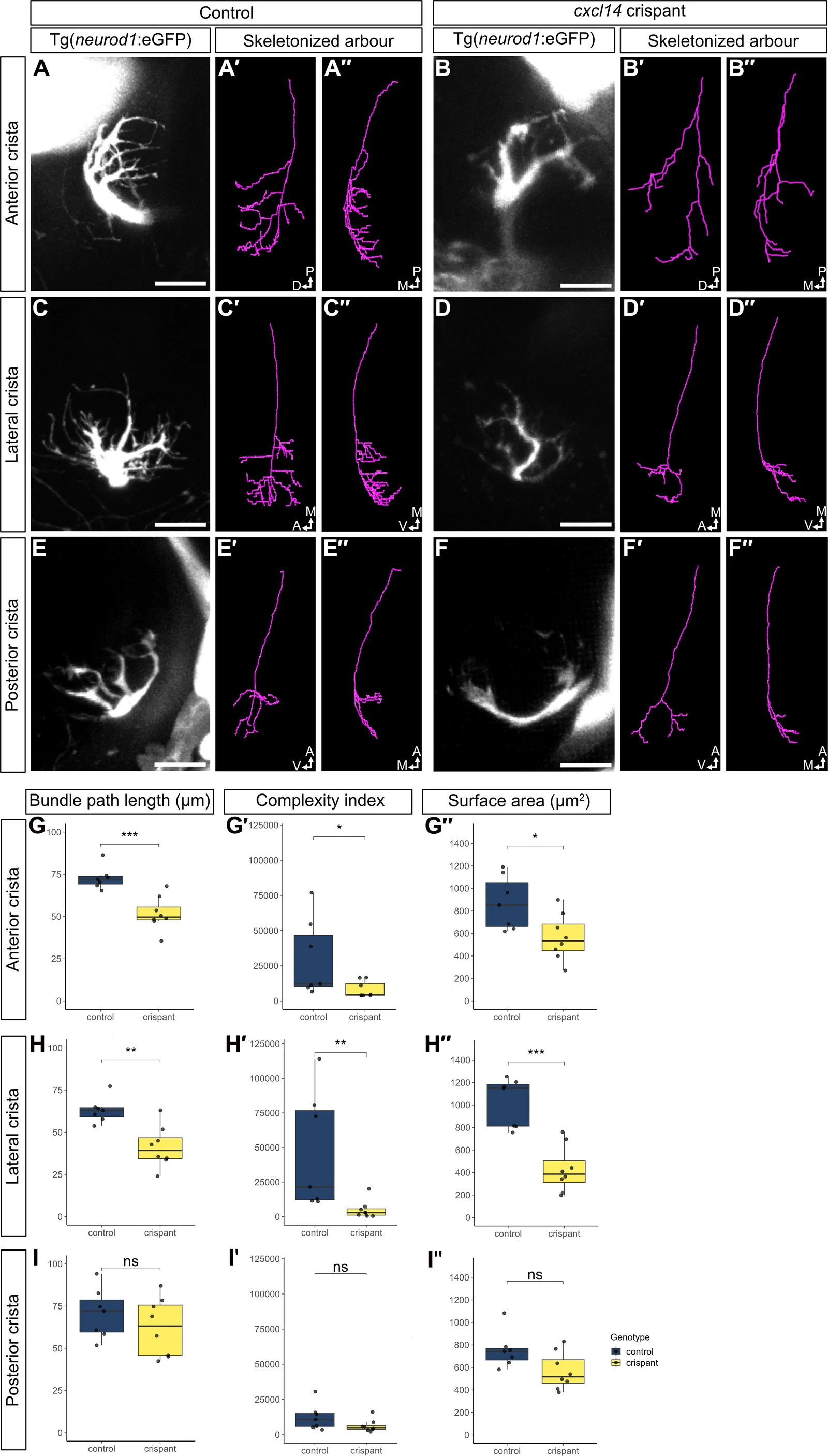
***Cxcl14* depletion causes a reduction in axonal arbour morphology complexity in innervation sites of cristae. (A–F″)** Representative examples of control and *cxcl14* crispant axonal arborizations in anterior crista, lateral crista, and posterior crista innervation sites. Each panel depicts the confocal max projection of axonal terminations visualized by TgBAC(*neurod1:eGFP*)^nl1^ and a skeletonized reconstruction of the axon from two different views (magenta). Scale bars = 10 µm. **(G–I″)** Boxplots showing the quantification of axon bundle path length (µm) (anterior crista = 72.8 ± 2.55 for controls and 51.0 ± 3.48 for *cxcl14* crispants, ***P = 0.0006; lateral crista = 63 ± 2.79 and 41.3 ± 4.29, **P = 0.004; posterior crista = 70.6 ± 5.59 and 62.4 ± 6.05, P = 0.4), axonal arbour complexity index (ac = 29,979 ± 10,367 for controls and 8,084 ± 2,031 for *cxcl14* crispants, lc = 46,329 ± 15,924 and 5,112 ± 2,305, **P = 0.002; pc = 12,354 ± 3,515 and 6,230 ± 1,571, P = 0.15) and surface area (µm^2^) (ac = 870 ± 89 for controls and 567 ± 72.3 for *cxcl14* crispants,*P = 0.029; lc = 1,022 ± 82 and 429 ± 72, ***P = 0.0006; pc = 754 ± 60.7 and 567 ± 57.8, P = 0.07) in control (dark blue) and *cxcl14* crispant (yellow) embryos for each crista. *N* = 7 controls/8 *cxcl14* crispants from two independent experiments. Values are given as mean ± SD. Mann–Whitney test was performed in all comparisons.

We analyzed afferent SAG axon arborization in the cristae where axonal terminals are well separated from somas, facilitating their imaging. We traced and 3D-reconstructed axonal terminal arborizations in anterior, lateral, and posterior cristae (ac, lc, and pc, respectively) (Fig. 7, A–F″) using the Single Neurite Trace (SNT) plugin in Fiji. In controls, fasciculated bundles were sent from somas of the SAG to each specific crista, and when they reached them, axons turned at an angle greater than 90° and ramified to innervate each HC. In crispant embryos, axon bundles were still growing toward the cristae. Nevertheless, the overall ramifications were composed of fewer branches.

We then quantified several parameters of axonal terminals to assess their complexity (Fig. 7, G–I″). Regarding the ac, branches were misrouted in 50% of crispant embryos (*N* = 8), resulting in some axons being defasciculated from the bundle and the fasciculated bundle being shorter (Fig. 7, B and B″). In the case of lc, there were cases in which axons were hard to distinguish, as they were very few and thinner than in controls (data not shown). The length of the fasciculated bundle was also reduced in crispant embryos (Fig. 7, G–I), compared with controls (*N* = 7), and the axonal complexity index (i.e., how intricate an axonal arbor is) and axonal surface area were dramatically reduced, indicative of a reduction in innervation capability (Fig. 7, G–I″). Surprisingly, pc was less affected than the other cristae, showing a tendency toward a lower complexity index and smaller surface area. These differences were not statistically significant (Fig. 7, I–I″).

**Figure S3.**
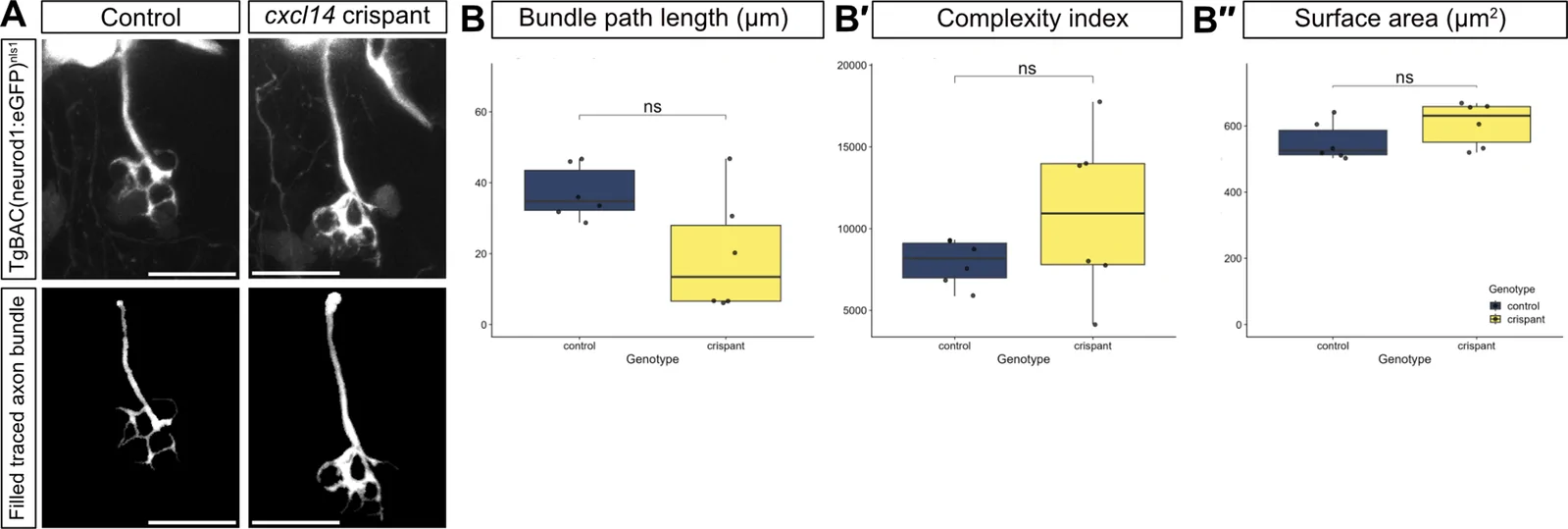
***cxcl14* depletion does not affect axonal innervation morphology of neuromasts. (A)** Representative examples of control and *cxcl14* crispant axonal arborizations in neuromast IO4. The upper panels depict the confocal max projection of axonal terminations visualized by TgBAC(*neurod1:eGFP*)^nl1^ and a volume reconstruction is depicted in the lower panels. Scale bars = 20 µm. **(B–B″)** Box plots showing the quantification of axon bundle path length (µm) (37.12 ± 7.53 µm for controls and 19.49 ± 16.6 µm for crispants, P = 0.1), axonal arbour complexity index (7924.27 ± 1416.02 for controls and 10918.68 ± 5083.88 for crispants, P = 0.39) and surface area (µm^2^) (551.64 ± 57.37 for controls and 607.02 ± 66.48 for crispants, P = 0.07) in control (dark blue) and *cxcl14* crispant (yellow) embryos for each crista. *N* = 6 controls/6 *cxcl14* crispants from two independent experiments. Mann–Whitney test was performed in all comparisons.

We also quantified the same parameters in the axon bundle targeting one of the cranial neuromasts (IO4) in controls and *cxcl14* crispants (*N* = 6 and *N* = 6, respectively) to assess if LL innervation was also affected by the disruption of Cxcl14. No significant differences were found (Fig. S3), suggesting that the LL innervation does not depend on Cxcl14 signaling.

Together, these results show that Cxcl14 is involved in axonal arborization in cristae and that the lack of the chemokine results in less complex branches covering a smaller area.

### The vestibuloacoustic response is impaired in *cxcl14* crispant embryos

To test whether the phenotypes observed at a cell-resolution level were affecting the overall vestibuloacoustic system’s responses, we performed a behavioral test using the DanioVision observer chamber. We tested the startle response following a tapping stimulus in 5 dpf *cxcl14* crispant and control larvae. The startle response is an escape mechanism that appears from 5 dpf onward, characterized by a rapid C-bend of the body followed by swimming. It is mainly mediated by the vestibuloacoustic system, which senses vibrations; Mauthner cells integrate the sensory input and initiate the motor response (Hale et al., 2016; Tabor et al., 2014).

**Figure 8.**
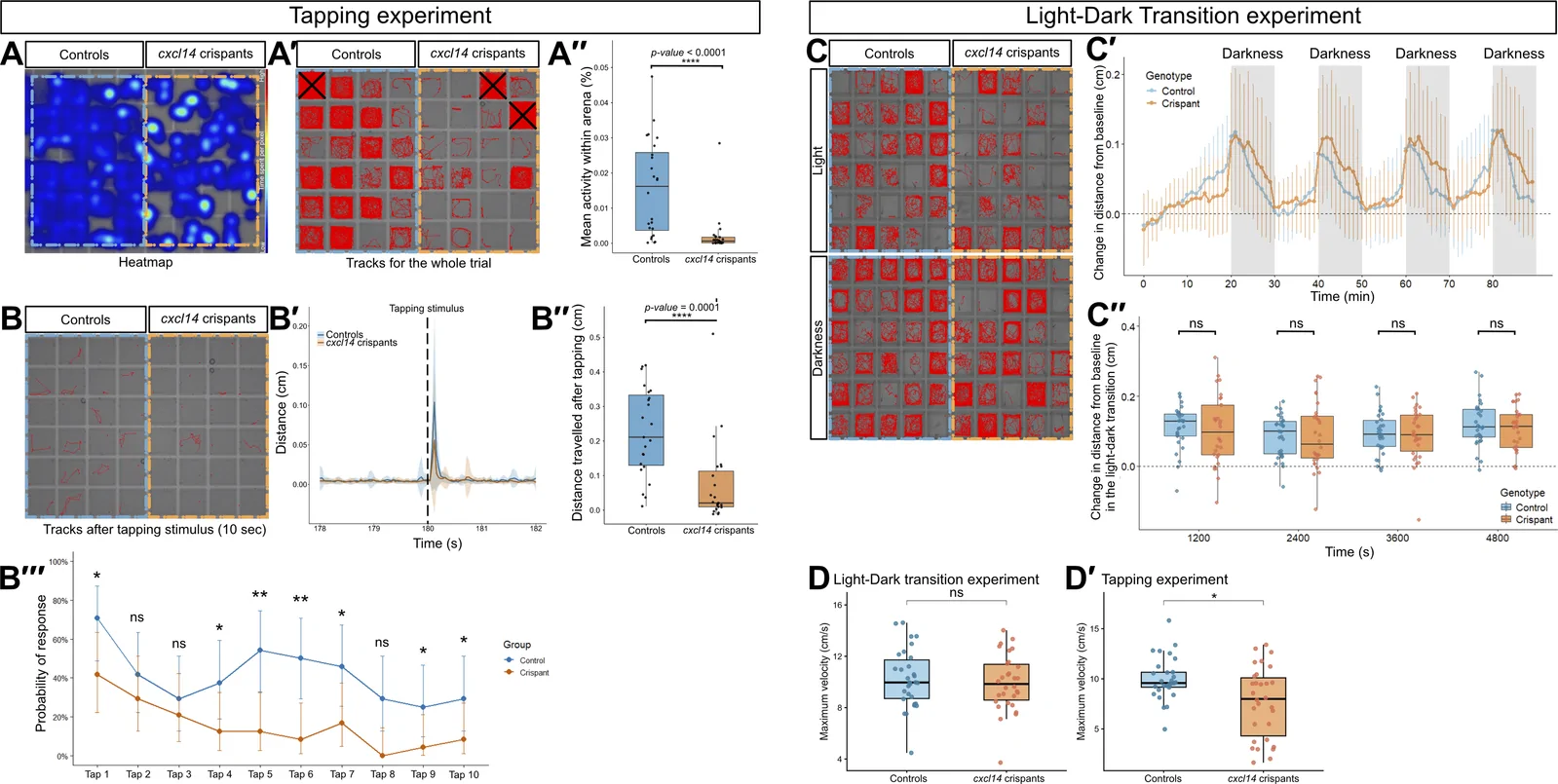
**Startle response after acoustic stimuli is reduced in *cxcl14* crispant 5dpf larvae. (A–D) **Results corresponding to the tapping experiment. **(A)** Heatmap of the total time spent per pixel during the whole tapping experiment. Colder colors in the scale represent a lower amount of time that the larvae have stayed on a pixel, while hotter colors represent a higher amount of time. **(A′)** Tracking of the path (red) followed by larvae on their corresponding wells during the tapping experiment. Wells marked with an X were discarded for further analysis due to faulty tracking. **(A″)** Boxplot representing the mean individual activity of control (blue) and *cxcl14* crispant (orange) larvae, represented as the % of the total area of the arena, which corresponds to each well. Individual values are represented as dots. Control group = 0.016 ± 0.013, *cxcl14* crispants = 0.002 ± 0.006. ****P = 0.0000029. **(B)** Tracking of the path (red) followed by larvae in the period of 10 s after a tapping stimulus. **(B′)** Example of the distance moved by larvae before and after stimulus. Line plot of the distance moved by the larvae 2 s before stimulus and 2 s after tapping. The stimulus is marked with a dotted line at the second 180. **(B″)** Startle response strength. Boxplot representing the individual average of the distance moved 1 s after tapping stimuli normalized by the baseline of each larva (dots represent individual values for each larva). Control group = 0.227 ± 0.026; *cxcl14* crispants = 0.074 ± 0.036. ***P= 0.00012. **(B‴)** Line plot representing the probability of response to each tap (percentage). *P value < 0.05, **P value < 0.01. **(C–D′)** Results corresponsing to Light-Dark Transition experiment. **(C)** Example of a tracking of the path (red) followed by larvae during light (upper panels) and darkness (inferior panels) periods. **(C′)** Line plot depicting the change in distance from baseline (cm) over time (min) in controls (blue) and *cxcl14 *crispants (orange). Darkness periods are depicted by grey rectangular areas. **(C″)** Boxplot representing the change in distance from the baseline (cm) travelled by larvae after each transition from light to darkness. X-axis depicts the exact timepoint (sec) in which each transition happens. Values have been normalized and the baseline is 0. controls = 0.11 cm ± 0.06, 0.09 ± 0.06, 0.09 ± 0.06, 0.12 ± 0.06; *cxcl14* crispants = 0.10 ± 0.09, 0.09 ± 0.09, 0.09 ± 0.07, 0.10 ± 0.07 (Mean ± S.D.). **(D and D′)**. Box plot representing the maximum velocity reached by each larva during the whole light-dark transition experiment or the whole tapping experiment, respectively. Mean for the light-dark transition experiment: Control group = 1′.26 ± 2.25 cm/s, *cxcl14* crispants = 9.94 ± 2.16 cm/s. P = 0.66. Mean for the tapping experiment: Control group = 9.98 ± 2.08 cm/s, *cxcl14* crispants = 7.65 ± 3.46 cm/s. *P = 0.016. Values are given as mean ± SD. Mann–Whitney test was performed for the activity within arena comparisons, and a linear mixed-effects model was used for the startle response strength comparisons and for the probability of response. Wilcoxon test was used for pair comparisons in C″ and D and D′. Tapping experiment: *N* = 23 controls/22 *cxcl14* crispants. Light-dark transition experiment: *N* = 30 controls/30 *cxcl14* crispants.

Based on the tracking for the whole experiment that includes all tapping stimuli, *cxcl14* crispant larvae moved less than their counterpart control siblings (Fig. 8, A–A″) (Video 6). We visually analyzed the tracking recording and observed that after each tapping, a smaller number of *cxcl14* crispant larvae showed a startle response (the average value for all the tappings of *cxcl14* crispant larvae that moved after tapping was 4.2 out of 22 larvae ± 2.39) in comparison with control larvae (14.9 out of 23 larvae ± 2.56) (Fig. 8 B). The intensity of the startle response, represented as the distance moved after the stimulus, was also diminished in *cxcl14* crispants (Fig. 8, B′ and B″). This resulted in a lower distance moved by *cxcl14* crispants compared with controls. We also calculated the response probability of each larva to each tap, which showed a lower probability of responding from crispant larvae compared with controls (Fig. 8 B‴).

**Video 6.** **Time-lapse of confocal microscopy showing the disassembly of SAG–pLL axon contacts from 26 to 33.5 hpf (stills of this time-lapse are shown in Fig. S1).** Scale bar = 10 µm. Frames were obtained every 15 min and 45 s.

However, these differences could be derived from a locomotor impairment instead of a vestibuloacoustic affectation. To test locomotor capacity, we added a light/dark transition test, which assesses motor responsiveness independently of a mechanosensory input (Fig. 8, C–C″). Similarly to the first test, crispant larvae tended to move less during the accommodation period (light) (Fig. 8 C). Nevertheless, after the sudden transition to darkness, both groups, control and crispant, increased their movement (Fig. 8 C′). When analyzing the distance moved a second after the transition to darkness, no significant difference was observed between the two groups (Fig. 8 C″), indicating that crispant larvae responded to this visual stimulus in a similar fashion. Moreover, the maximum peak velocity that individual larvae could reach during the light/dark transition test shows no statistically significant differences as well (Fig. 8 D), suggesting that locomotive capacity is not affected.

Although not shown in the tracking, we could observe that some *cxcl14* crispant larvae fell laterally when they were not swimming, with large periods of immobility, a sign of lacking stationary balance (*N* = 15/82). No controls displayed this behavior. Moreover, 4 out of 24 larvae analyzed in the light-dark experiment displayed postural dysregulation when swimming (Video 7). Therefore, these results suggest that the lack of *cxcl14* has an effect on vestibuloacoustic function, but not locomotor activity, in 5 dpf larvae.

**Video 7.** **Video obtained in the Noldus DanioVision observation chamber showing examples of the basal activity in control and *cxcl14* crispant larvae (5 dpf) during the accommodation period in the behavioral test.** Time is depicted as mm:ss. The images were obtained at 25 fps.

## Discussion

Our findings uncover two general principles of broad relevance. First, peripheral sensory circuits utilize pioneer-based cellular scaffolds analogous to those described in the CNS, challenging the prevailing view that inner ear circuitry derives exclusively from otic-derived neurons. Second, our work reveals chemokines as bona fide regulators of axon navigation during development, extending their functional repertoire beyond immune and inflammatory contexts. In particular, we reveal a new role of the little-studied Cxcl14 chemokine in directed axonal growth and HC activity.

In the mouse inner ear, several molecules have been proposed to guide auditory and vestibular afferents to the hair cells located in the sensory epithelium at late developmental stages (Hashimoto and Heinrich, 1997; Fritzsch et al., 2005; Kim et al., 2016; Jung et al., 2019; Cantu-Guerra et al., 2023). However, early developing axons extend to sensory regions before hair cells are fully differentiated (Hemond and Morest, 1992), suggesting that early secreted OV trophic factors fulfill this guidance role. In particular, the chemokine MIF was found to promote the growth of chick and mouse SAG axons *in vitro* (Bianchi et al., 2005), but its expression was not found in the developing sensory patches *in vivo*. Thus, how the first axons are directed to that specific region to innervate the HCs has not been resolved.

Here, we show that pioneer axons are extended by a population of pioneer cells of non-otic origin sharing markers with aLL progenitors. Both aLL and otic progenitors derive from a common OEPD domain expressing *pax2* and *pax8*, and although aLL and otic progenitors segregate over time, it is plausible that at early stages an uncommitted population of cells serves as pioneers for SAG and aLL ganglion development. Whilst the role of pioneers in pLL has been investigated (Gompel et al., 2001; Pujol-Martí and López-Schier, 2013; Woodruff et al., 2025), this is the first report of a role of pioneer axons in inner ear development, showing that pioneer axons serve as scaffolds for neuroblast migration and organization of SAG lobes.

The use of the zebrafish has also permitted us to image at high spatiotemporal resolution how pioneer SAG axons extend toward HCs. As shown in other contexts (Ma and Tessier-Lavigne, 2007; Lewis et al., 2013; Kalil and Dent, 2014), axons emitted dynamic branches that eventually were retracted, but SAG pioneer axons and LL axons established transient contacts that were maintained for longer time periods. It has recently been proposed that axon–axon interactions help to establish a correct somatosensory map in *Drosophila* (Galindo et al., 2023). When cIV neurons were ablated, axons of cIII invaded new territories, suggesting that transient axon–axon interactions might have an inhibitory effect on their growth and targeting area. Therefore, it is plausible that transient SAG and LL axon–axon interactions are a developmental mechanism to finally segregate both circuits that develop in proximity. Thus, the correct growth and targeting of SAG pioneer axons seems fundamental for the precise contact with pLL axons, allowing the posterior segregation of the two distinct axons.

**Figure 9.**
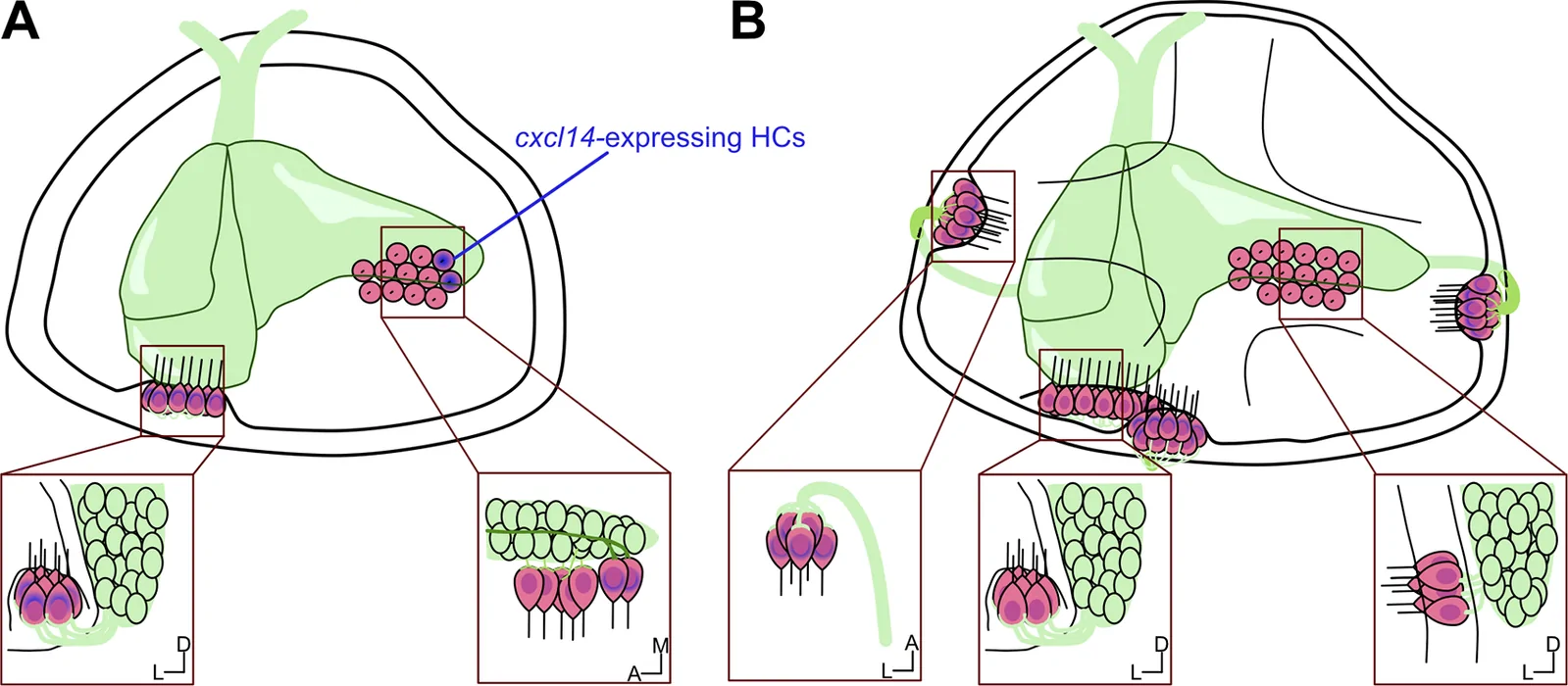
**Schematic representation of the spatial relationship between neurons and HCs**. **(A)** Schematic drawing of a lateral view of a 36 hpf otic vesicle (OV) depicting neurons (green) and maculae HCs (pink). Inset zooms show a detail of the innervation of the two sensory patches. *Cxcl14*-expressing HCs are depicted in blue. **(B)** Schematic representation of a 48 hpf OV, depicting SAG neurons and axons (green) and cristae and maculae HCs (pink). Insets depict details of the innervation of the sensory patches. *Cxcl14*-expressing HCs are depicted in blue.

Cxcl14 expression appears in first-born HCs and is restricted to a few HCs in macula and cristae, also expressing *cabp1b*. Expression appears and disappears first in the macula compared with cristae since these latter sensory patches differentiate afterward. This highly dynamic and transient expression pattern of *cxcl14* made us hypothesize that its function could be related to early innervation by pioneers. Interestingly, its expression in the pm was restricted to only the first two HCs that differentiated in this sensory patch, the tether cells, suggesting a difference between the two first-differentiated HCs and the rest. Thus, *cxcl14* serves to promote the posterior navigation of SAG pioneer axons to correctly target the first HCs of the pm. On the contrary, the later-born HCs of the pm are innervated by non-pioneer neuroblasts from the posterior lobe of the SAG, whose somas are in close contact with the macula and whose axons do not navigate or extend long distances to find the HCs (Fig. 9). Regarding the am, axons grow beneath the inner ear and turn dorsally to innervate the HCs. Although new HCs differentiate in maculae and cristae, *cxcl14* expression disappears, suggesting that it is not required for their function or their innervation at later time points. Expression in cristae at 5 dpf remains in supporting/precursor cells, which could be related to a later role in regeneration, as its upregulation has been detected by scRNA-seq analysis in regenerating neuromasts (Baek et al., 2022). The observed loss of directional growth of SAG pioneer axons to posterior HCs in *cxcl14-*depleted embryos, together with the reduction of axonal arborization complexity and area covered by axons, aligns with our hypothesis. Furthermore, in overexpression experiments, some axons were misrouted and extended to ectopic *cxcl14*-expressing cells, suggesting a role of the chemokine in guiding certain axons.

As the expression of *cxcl14* in entry points initiated concomitantly with the extension and fasciculation of central axons, and in *cxcl14*-depleted embryos, central projections failed to correctly enter the hindbrain and displayed navigation, fasciculation, and turning errors, we also propose that *cxcl14* regulates central projection growth to the hindbrain. It would be interesting to assess whether in other vertebrates *cxcl14* is also present in entry sites of central projections and regulates these projections. Finally, the nature of the cells expressing *cxcl14* in the entry points is still elusive. They might have a neural crest origin, as neural crest cells migrate in two streams adjacent to the otic placode and, in the dorsal root ganglion, neural crest–derived boundary cap cells regulate the entry and exit of sensory and motor axons (An et al., 2002). Further studies should investigate this question.

Besides the effect of the chemokine on axonal growth and targeting, additional roles in HC physiology cannot be excluded. We found reduced HC activity, and Otoferlin was not properly distributed towards the base of HCs in *cxcl14*-depleted embryos, suggesting a disruption of synapse vesicle trafficking. The HC phenotype could be a direct effect of the lack of *cxcl14* or an indirect effect from improper axonal innervation. In mice, afferent contacts preceded presynaptic ribbon attachment in the base of HCs (Michanski et al., 2019), suggesting a possible correlation between innervation and HC synaptic activation. Nevertheless, studies in zebrafish LL neuromasts demonstrated that HCs were active in larvae that lacked innervation (López-Schier et al., 2004). Therefore, c*xcl14* could be affecting both HC activity and axon growth and targeting. In this regard, recent work in the somatosensory cortex revealed that Cxcl14 is required for regulating SBC axonal morphology and excitability (Iannone et al., 2024). The lack of a known receptor for Cxcl14 also impedes the study of a possible autocrine effect in HCs. Moreover, no specific quantification of the signaling distance or effective diffusion for Cxcl14 is reported. For CXCL12, the effective tissue diffusion was mathematically modelled to be small (around 5–25 μm^2^/s); thus, it is plausible that Cxcl14 has a relatively short-distance signaling range as well (Venkiteswaran et al., 2013). Previous studies also reported an autocrine signaling mode in tissue grafts (Augsten et al., 2009).

As both HCs and axonal innervation were affected by the lack of *cxcl14*, we tested if the implication of the chemokine was translated into a general malfunctioning of the vestibuloacoustic system. Indeed, we found an impairment in the stationary balance of 5 dpf *cxcl14* crispant larvae, together with a reduced startle response when larvae were submitted to acoustic stimuli. The reduction in general locomotor activity could be explained by the lack of stationary balance (Riley and Moorman, 2000; Kim et al., 2014). Our behavioral test cannot distinguish between a defect specifically affecting the inner ear circuitry and an alteration of the LL system. However, taking into consideration that we did not find gross defects in axons targeting neuromasts and that *cxcl14* is not expressed in neuromast HCs but in surrounding cells, we conclude that the observed behavioral deficits are mainly caused by inner ear axonal phenotypes. Altogether, the results suggest that the correct function of the statoacoustic sensory system relies on the chemokine *cxcl14*.

Due to the evolutionary conservation of *cxcl14* in inner ear HCs in all vertebrates analyzed so far, our work in zebrafish sets the basis for future functional studies in other vertebrates. Interestingly, recent scRNA-seq datasets identified *cxcl14* to be enriched in mouse and human utricles (Wang et al., 2024), in regenerating type II HCs in mouse and human utricles (Luca et al., 2025), in tall HCs of the postnatal day 7 chick cochlea, and in chick regenerating utricle HCs (Ku et al., 2014; Janesick et al., 2022). As our work provides functional evidence for the requirement of Cxcl14 in HC activity and setting the pioneer neural circuit, we speculate that Cxcl14 induction in regenerating HCs might be necessary for reinnervation of HCs after injury. Nevertheless, further studies are needed to confirm this hypothesis in regenerating tissues.

Ultimately, these findings fundamentally reshape our understanding of peripheral neurodevelopment by demonstrating that inner ear circuit assembly relies on pioneer axonal scaffolds and expands the functional repertoire of chemokines into essential developmental axon guidance cues.

## Materials and methods

### Fish maintenance and husbandry of transgenic lines

Zebrafish embryos and adults were maintained and handled according to standard procedures at the aquatic facility of the Parc de Recerca Biomèdica de Barcelona, in compliance with the guidelines of the European Community Directive and the Spanish legislation for the experimental use of animals, as previously described (Westerfield, 2000). Stable transgenic lines were kept by means of alternate outcross with WT (AB/Tü) and incross, generation after generation. Embryos were kept in the dark at a temperature of either 23 or 28′5°C in Danieau’s solution at a confluence of 50 embryos per plate until the desired stages.

Embryos older than 24 hpf were maintained in embryo medium (Danieau’s solution) containing 1% 1-phenyl-2-thiourea (Sigma-Aldrich) to inhibit pigment formation. Embryos were staged as previously described (Kimmel et al., 1995). All the embryos used that were still in the chorion were manually dechorionated.

For this study, we have used the following lines: *TgBAC(neurod1:eGFP)*^*nl1*^ (Obholzer et al., 2008), *Tg(neurod1:kikume)* (generated in the lab, Bañón and Alsina, 2023), *Tg(brn3c:GFP)*^*s356t*^ (Xiao et al., 2005).

### Microinjection

Long and very thin injecting needles were pulled in an electrophysiology puller (Sutter Instruments model P-97) with the following protocol: *P* = 200; HEAT = 566; PULL = 90; VEL = 70; TIME = 80. The tip of the needle was bevel-broken using forceps, and the needle was loaded with an injection solution. Embryos were injected with 1 or 2 nl into the cell in one-cell-stage embryos using an Eppendorf FemtoJet 4i microinjector.

For CRISPR-Cas9 KO experiments, a mix containing 1.33 μl of each duplexed gRNA (3 dgRNA [crRNA+tracrRNA] in total, stock concentration 25 µM), 0.5 μl (stock concentration 62 µM) of Alt R S.p.HiFi Cas 9 Nuclease v3 (#1081060) and 0.5 μl of nuclease-free water. Cxcl14 crRNAs were designed with the CHOPCHOP platform (https://chopchop.cbu.uib.no/) following published guidelines (Wu et al., 2018; Hoshijima et al., 2019). crRNA sequences targeting *cxcl14* for CRISPR-Cas9 KO: 5′-CAT​TTA​TTC​GCT​CAA​CAC​AG-3′, 5′-GCA​CTA​GGA​ACC​TTG​TCA​AG-3′ and 5′-GGG​GAT​GAA​TCG​CTG​TAG​TA-3′. Scrambled crRNAs (randomization of antibiotic resistance sequences) for CRISPR-Cas9 experiments: 5′-GGC​TCC​TTG​CTC​GCG​ACT​TG-3′, 5′-GGC​GAC​CGT​GGC​CGG​AAC​GA-3′, and 5′-GCC​TGC​GCG​TAC​CGA​CTC​GG-3′. All crRNAs were ordered from Integrated DNA Technologies (IDT). Each crRNA was mixed with Alt-R tracrRNA (1:1, IDT) to obtain duplexed gRNA.

For Tol2 injections (hsp70I:cxcl14-IRES-CAAX-GFP), a mix of 1 μl of Tol2 RNA at 175–200 ng/μl, 3 μl of plasmid at 50 ng/μl, and 6 μl of dH_2_0 was prepared. 1 nl of this mix was injected into the cells of one-cell-stage embryos.

### Genotyping *cxcl14* F_0_ KO (crispants)

Genomic DNA extraction of embryos was performed using the N-Amp extraction kit (XNAT2 Extract-N-Amp Tissue PCR Kit XNAT2-1KT). PCR protocol was as follows: Fw primer: 5′-ACA​GAG​TTT​ATC​ATC​AAA​CCG-3′, Rv primer: 5′-ACT​TTA​ACT​TCG​CAA​ATA​TGC​C-3′. An initial denaturation at 95°C 3 min; 35 cycles of 94°C 30 s; 58°C 30 s; 72°C 30 s; 72°C 7 min; hold at 4°C. Heteroduplex mobility assay was performed (electrophoresis was carried out on a 3% agarose gel, at 120V for 1 h 30 min), followed by Sanger sequencing of the samples to confirm mutations.

### Photoconversion

Photoconvertible *Tg(neurod:kikume)* embryos were maintained in the dark to avoid spontaneous photoconversion. Circular ROIs of the desired size were drawn using the Zeiss ZEN 3.11. The bleaching option of the software was employed for photoconversion. UV 405 laser power was set to 10% and conducted in 3–4 iterations at a speed of 200 Hz. An image prior to and after photoconversion was obtained, enabling confirmation of correct photoconversion.

### Photoablation

An SP5 inverted Leica Multiphoton confocal microscope was used with a Mai Tai multiphoton activated (Mai Tai BB DeepSee [Spectra Physics] tunable [710–990 nm] pulsed laser), humidity 4%, and temperature 20°C. A BS/RLD mirror and SP715 filter were used with a 910 nm laser at 42% power at 20× zoom with an HC PL APO 20×/0.75 immersion objective (506191; Leica) because no ROIs can be used in this confocal in multiphoton mode. PMT was employed, and the pinhole was kept completely open (600 nm). Scans ranged from three to six until a bubble formed (indicating destroyed tissue). Scan speed was 200–400 Hz, frame size was 1,024 × 512, bidirectional scanning was on, with line and frame average 1.

### 4-Di-2-ASP staining and injection into the OV

Larvae were maintained as described until 72 hpf. Dechorionated larvae were anesthetized using 42 μl of Tricaine (4 mg/ml, MS-222) per 1 ml of embryo medium and placed in a lateral position on top of a 3% agarose-coated petri dish. Using the same setup as the one described in the microinjection section, 5 nl (20 ng/ml) of 4-Di-2-Asp (D3418; Sigma-Aldrich) was injected into one of the otic vesicles of the embryos. To label hair cells from neuromasts, embryos were then incubated in a 1:1,000 dilution of 4-Di-2-Asp (1 μl of 20 mg/ml in 1 ml of Danieau’s solution) shaking for 40 min. After three washes with Danieau’s solution, the embryos were returned to the incubator until the imaging step.

### *In situ* hybridization of whole-mount embryos

The embryo whole-mount *in situ* hybridization protocol was adapted from Thisse and Thisse (2008). Antisense riboprobes were generated by *in vitro* transcription using DIG or FLUO-dNTPs of cloned cDNA or directly from PCR-amplified fragments, using primers containing the promoter sequence of T7 or SP6 sequences at the 5′ ends of the reverse primer. Fixed embryos were dehydrated with methanol and stored at −20°C. After rehydration, permeabilization with proteinase K (diluted 1:1,000 from a stock of 10 mg/ml, Invitrogen), embryos were incubated with the hybridization buffer (50% FAD, 25% SSC, 250 mg torula RNA, 1,25 mg heparin, and Tween 0.1%) at 70°C for 2 h. Incubation with the riboprobes diluted in hybridization buffer at a concentration of 0.5 ng/μl (DIG-labeled probes) or 2 ng/μl (FLUO-labeled) was done overnight at 70°C. Embryos were washed and incubated with a blocking solution (2% blocking reagent [11096176001; Roche] 10% neutralized goat serum in 1x MABT) for 1 h. Incubation with anti-FLUO-POD (1:400), anti-DIG-POD (1:1,000), anti-FLUO-AP (1:2,000), or anti-DIG-AP (1:2,000), respectively, was performed O/N at 4°C. Fluorogenic *in situ* hybridization probes were developed with TSA Fluorescein and Cy3 (NEL753001KT; Akoya), respectively, for 1 h. Chromogenic *in situ* hybridization probes were developed with NBT/BCIP. Signal development was assessed visually, time ranging in a probe-dependent fashion. After stopping the reaction with dH_2_O, embryos were kept in PBS with 0.1% Tween-20 (PBST)/glycerol 1:1 and flat-mounted for imaging.

### *In situ* hybridization chain reaction of whole-mount embryos

Zebrafish embryos at selected stages were fixed overnight in 4% PFA at 4°C, dehydrated through a methanol series (25%, 50%, 75%, and 100%), and stored at −20°C until use. After rehydration, embryos were permeabilized with proteinase K (10 μg/ml in PBST) for 5 min at 18 hpf, 15 min at 36 hpf, or 30 min at 72 hpf, followed by post-fixation in 4% PFA for 20 min at room temperature. HCR Gold RNA-FISH was performed according to the manufacturer’s instructions (Molecular Instruments). Custom probe sets targeting *greb1l* (X2), *cabp1b* (X2), and *cxcl14* (X1) were used with corresponding amplifiers (X1–488, X2–546). Hybridization was carried out overnight, followed by overnight incubation with amplifiers. Embryos were imaged using an LSM990 Zeiss upright confocal microscope. A water-dipping 20× objective was used (Zeiss, W Plan-Apochromat 20×/1.0 DIC VIS – IR).

### Embryo immunostaining

Embryos were fixed at the desired stage in 4% formaldehyde, washed three times for 5 min with PBST 0.1%, and permeabilized with proteinase K (diluted 1:1,000 from a stock of 10 mg/ml, Invitrogen). Larvae were washed two times for 10 min in PBT 0.1%, post-fixed with 4% PFA, and blocked in 10% neutralized goat serum and 2% bovine serum albumin in PBST for 2 h at room temperature. Embryos were incubated overnight at 4°C with antibodies diluted in blocking solution. Primary antibodies used: rabbit anti-GFP (1:400; TP401; Torrey Pines), mouse anti-Otoferlin (clone HCS-1, 1:25, Developmental Studies Hybridoma Bank, AB_10804296). After washing with PBST, embryos were incubated with secondary antibodies conjugated with Alexa Fluor 488, 594, or 633 (1:500 dilution in blocking solution; A-11029, A-21206, A-11037, A-21070, or A-21053; Invitrogen).

### Confocal imaging

Live zebrafish embryos were anesthetized with MS-222(Tricaine) at the desired stage. Fixed or live zebrafish embryos were mounted 45° from completely lateral or dorsally in 0.8% low melting point agarose (90002155; Ecogen) on a 35-mm glass-bottom Petri dish (P35G-1.5-14-C; Mattek). The agarose was covered with 3 ml of PBST (if fixed) or Danieau’s solution with Tricaine (if live). Images and time series were acquired with the LSM980 inverted confocal or LSM990 upright confocal systems (Zeiss). Each channel was acquired rastering by line, bidirectionally, using the 40× immersion objective (420862-9970-799; Zeiss, NA 1.2) with silicone oil. For the LSM990 system, a water-dipping 20× objective was used (Zeiss, W Plan-Apochromat 20×/1.0 DIC VIS – IR). A z-step of 1 µm was selected. The image format was 1,024 × 1,024 or 2,048 × 1,024, and the scan speed ranged between 1.89 and 3.77 s/frame. 488 and 561 nm laser excitation varied from 1 to 10% for *in vivo* acquisitions and up to 30% for fixed samples. The same settings, laser power, and gain were maintained among embryos of the same experimental unit. Multi-position experiments (time series with multiple embryos) included up to 10 embryos separated by a time frame no longer than 20 min. Airyscan function was used for imaging hair cells labelled with 4-Di-2-ASP and Otoferlin antibody (Developmental Studies Hybridoma Bank, AB_10804296) to obtain higher spatial resolution images. For the live imaging, the temperature was set to 28.5°C using an environmental chamber.

### Image processing

FIJI software was used to analyze confocal images. Image brightness and contrast were adjusted linearly. MAX projections of the whole z-stack or specific slices were performed. Gaussian blur, background subtraction (50-pixel radius), and/or a minimum filter were applied to images for visualization purposes only. Data quantification was obtained from raw images. When comparing signal intensity in different conditions (i.e., 4-Di-2-ASP in control and crispant embryos), the same values of linear modifications were applied to all images. 3D drift correction (Fiji plugin) was applied to time series acquisitions to correct tissue displacement at a pace of 4–10 µm/h.

### 3D reconstruction and quantification of axons

FIJI SNT from Neuroanatomy (https://imagej.net/plugins/snt/index) was used to trace axonal arborizations of z-stack images from 48 hpf embryos. ROIs containing an axon bundle directed to each crista were defined, starting from the most medial slice, where the bundle started from the somas to the tip of the most lateral branch. The bundle was analyzed as a single axon. The bundle was traced up to the initiation of branches, and each branch was traced individually. Quantifications were performed using the analysis tools of SNT (cell-based analysis).

### Otoferlin quantification

From each crista analyzed (Fig. 7), lines were hand-drawn using the Fiji line tool, from the most apical tip of a hair cell to the base using the channel for HC membranes (*Tg(brn3c:GFP)*^*s356t*^*)*. Six random HCs were selected from each crista. The intensity of the Otoferlin signal was then plotted using the plot profile function in Fiji, and the mean intensity of apical and basal regions was calculated using Excel. Comparisons and plots of the apical/basal ratio were performed using RStudio.

### Behavioral analysis

Fish were injected as previously described with either *cxcl14* or scrambled (control) gRNAs. Sibling larvae of both were kept at 28.5°C until 5dpf. Individual larvae were placed in wells of a 96-square-well plate containing 200 μl of embryo water to be acclimatized for 2 h before the behavior analysis (24 *cxcl14* crispant and 24 control larvae per plate). Behavior analysis was performed using the DanioVision Observation Chamber (Noldus Information Technology) linked with the EthoVision XT17 software. A steady flow of water was supplied to the chamber and maintained the larvae at 28.0°C during the whole recording. Camera resolution was set at 1,280 × 960, and the frame rate was set at 25 frames per second. Larvae were placed in the chamber for 30 min of habituation before starting the behavioral test. The experiment was conducted with the light on at an intensity of 5%. The experiment consisted of a startle response test elicited by 10 mechanical taps at 90-s intervals. The taps were generated using an inbuilt solenoid positioned at the base of the DanioVision chamber, with the stimulus intensity set to the highest level (level 8). For the light-dark sudden transition test, a period of 10 min of light was followed by 10 min of complete darkness. These two periods were repeated four times. These cycles of light-darkness were followed by 10 mechanical taps to test the startle response again to test whether comparisons could be made between different experiments.

EthoVision XT17 analyzes changes in pixels per frame. Activity within the arena was quantified as the percentage of pixels moving in each well. Distance travelled was quantified as the total movement of the center point detected for each larva. From raw individual tracking data, the startle response strength of each larva was calculated as the distance moved within the second after stimulus, normalized by the baseline distance moved per second during the 5 s before the stimulus (the baseline activity was subtracted from the distance moved during the startle stimulus). Then, the average and SD of control and crispant groups were calculated. Distance travelled per second was quantified by summing frame-to-frame displacements in 1 s (sample rate = 25 fps). Each individual was measured for all 10 taps, then normalized and averaged, and, finally, group means and SDs were calculated. For response probability measurements, a threshold of 3 cm/s was selected to determine that larvae were showing a burst in their swimming, indicative of a startle response. For each tap, a binary response was assigned to each larva (1 = surpassing the threshold, 0 = not surpassing the threshold). The number of positive larvae divided by the total number of larvae was calculated for both groups and for each tap. For the light/dark transition test, the baseline activity was calculated for each larva as the average distance travelled during the 10-minute period of adaptation. The baseline values were substracted from the distance moved after the transition to darkness. Then, group averages, SD and statistics were calculated.

### Statistical analysis and plots

Graphs were generated with GraphPad Prism 9 software or RStudio (version 2024.12.1). Shapiro–Wilk normality tests were performed to categorize the distribution of data into parametric or nonparametric tests. For non-normally distributed data, we used the Mann–Whitney–Wilcoxon test. In the analysis of startle response strength of the behavioral test, a linear mixed-effects analysis was used to test for effects from the different stimuli repeats. Values are expressed as mean ± SD.

### Single-cell RNA sequencing

The scRNA-seq datasets have been previously published (Olson et al., 2024; Woodruff et al., 2025) and deposited in the Gene Expression Omnibus database under accession codes: GSE266312, GSE264323, and GSE240721. The LL cluster was identified by expression of known LL markers (*hoxb5b*, *gfra1b*, and *hmx4*) but a lack of marker expression of other cranial sensory neurons (*irx1a* and *phox2bb*). This cluster was then subjected to unsupervised subclustering, which yielded 6 distinct subpopulations. To identify genes enriched in each subpopulation, differential expression (DE) analysis was performed with a minimum difference threshold set at 20% and logfc threshold = 0.2 using the “FindAllMarkers” function of Seurat. We then used top 10 DE markers to find the identity of each subcluster.

R code used in this study is available on GitHub: https://github.com/anechipor/Rumbo_et_al_2026.

### Online supplemental material

Fig. S1 and Fig. 6 show the disassembly of SAG and pLL axonal contacts. Fig. S2 corresponds to confocal images of SAG pioneer axons in control and *atoh1b-*downregulated embryos. Fig. S3 presents data on the morphology of aLL axonal bundle targeting neuromast O2 in control and *cxcl14* crispant embryos. Video 1 and Video 2 show the growth and branching of pioneer axon to the posterior region of the OV, supplementary data related to Fig. 2. Video 3 shows the crawling of neuroblasts along the pioneer axons to form the posterior lobe of the SAG (also related to Fig. 2). Video 4 and Video 5 show the examples of *cxcl14* crispant embryo (E1 and E2) from 22 to 25 hpf related to Fig. 4B. Video 7 presents data of examples of the basal activity in control and *cxcl14* crispants larvae (5 dpf) during accommodation period in the behavioral test.

## Supplementary Material

Review History

## Data Availability

The data supporting the findings of this study are available within the published article and its online supplementary material. The data underlying Fig. 1 are openly available in GitHub at https://github.com/anechipor/Rumbo_et_al_2026.
