# Supplementary material for: The Cxcl14 chemokine defines pioneer axon guidance and early circuit assembly in the inner ear
Source: J Cell Biol. 2026 Nov 11;226(2):e202604185. doi: 10.1083/jcb.202604185 (PMC13753874; doi:10.1083/jcb.202604185)
Supplement: Supplementary file 1 [file jcb_202604185_review_history.pdf]

JCB

The Cxcl14 chemokine defines pioneer axon guidance and early circuit assembly in the inner ear

Mireia Rumbo, Aitor Bañón-González, Alex Nechiporuk, and Berta Alsina

Corresponding Author(s): Berta Alsina, Universitat Pompeu Fabra

Review Timeline:

Submission Date:	2026-05-27
Editorial Decision:	2026-07-28
Revision Received:	2026-10-02
Editorial Decision:	2026-10-12
Revision Received:	2026-10-17

Monitoring Editor: Valerie Castellani

Scientific Editor: Dan Simon

Transaction Report:

(Note: With the exception of the correction of typographical or spelling errors that could be a source of ambiguity, letters and reports are not edited. The original formatting of letters and referee reports may not be reflected in this compilation.)

DOI: https://doi.org/10.1083/jcb.202604185

July 28, 2026

Re: JCB manuscript #202604185

Berta Alsina
Universitat Pompeu Fabra

Dear Dr. Alsina,

Thank you for submitting your manuscript entitled "The Cxcl14 chemokine defines pioneer axon guidance and early circuit assembly in the inner ear." The manuscript was assessed by expert reviewers, whose comments are appended to this letter. We invite you to submit a revision if you can address the reviewers' key concerns, as outlined here.

You will see that the reviewers feel that your study is well done and advances the current knowledge in the field. There are, however, two important concerns that would need to be addressed with new data in a revised study. Reviewer #3 notes a discrepancy between the claim that Cxcl14 is not expressed in lateral line hair cells and published scRNA-seq datasets which suggest that it is expressed. Similarly, Reviewer #1 asks to clarify which cell types in the inner ear express Cxcl14. Second, both Reviewers #1&3 feel that the behavioral analyses do not provide sufficient evidence that the mutant phenotype arises specifically from defects in vestibuloacoustic function. Reviewer #1 has a few additional questions which if you are able to answer could add interesting details and enhance the paper further. While we appreciate Reviewer#2's view regarding the suitability of this work for this journal, we and the other two reviewers agree that the paper is suitable for JCB.

While you are revising your manuscript, please also attend to the following editorial points to help expedite the publication of your manuscript. Please direct any editorial questions to the journal office.

GENERAL GUIDELINES:

Text limits: Character count for an Article is < 40,000, not including spaces. Count includes title page, abstract, introduction, results, discussion, and acknowledgments. Count does not include materials and methods, figure legends, references, tables, or supplemental legends.

Figures: Articles may have up to 10 main text figures. Figures must be prepared according to the policies outlined in our Instructions to Authors, under Data Presentation, https://jcb.rupress.org/site/misc/ifora.xhtml. All figures in accepted manuscripts will be screened prior to publication.

IMPORTANT: It is JCB policy that if requested, original data images must be made available. Failure to provide original images upon request will result in unavoidable delays in publication. Please ensure that you have access to all original microscopy and blot data images before submitting your revision.

Supplemental information: There are strict limits on the allowable amount of supplemental data. Articles may have up to 5 supplemental figures. Up to 10 supplemental videos or flash animations are allowed. A summary of all supplemental material should appear at the end of the Materials and methods section.

Statistical analysis: Error bars on graphic representations of numerical data must be clearly described in the figure legend. The number of independent data points (n) represented in a graph must be indicated in the legend. Please indicate whether 'n' refers to technical or biological replicates (i.e. number of analyzed cells, samples or animals, number of independent experiments). If independent experiments with multiple biological replicates have been performed, we recommend using distribution-reproducibility SuperPlots (please see Lord et al., JCB 2020) to better display the distribution of the entire dataset, and report statistics (such as means, error bars, and P values) that address the reproducibility of the findings.

Statistical methods should be explained in full in the materials and methods. For figures presenting pooled data the statistical measure should be defined in the figure legends. Please also be sure to indicate the statistical tests used in each of your experiments (both in the figure legend itself and in a separate methods section) as well as the parameters of the test (for example, if you ran a t-test, please indicate if it was one- or two-sided, etc.). Also, if you used parametric tests, please indicate if the data distribution was tested for normality (and if so, how). If not, you must state something to the effect that "Data distribution was assumed to be normal but this was not formally tested."

Please note that JCB now requires authors to submit Source Data used to generate figures containing gels and Western blots with all revised manuscripts. This Source Data consists of fully uncropped and unprocessed images for each gel/blot displayed in the main and supplemental figures. For assays performed using capillary electrophoresis and/or immunoassay-based detection, authors should instead provide the electropherogram graph(s) for each experiment, plotting fluorescence/chemiluminescence intensity vs. molecular weight/size. Please be sure to provide one Source Data file for each

figure gels, blots, and/or capillary electrophoresis assays along with your revised manuscript files. File names for Source Data figures should be alphanumeric without any spaces or special characters (i.e., SourceDataF#, where F# refers to the associated main figure number or SourceDataFS# for those associated with Supplementary figures). For traditional gels and blots, the lanes of the gels/blots should be labeled as they are in the associated figure, the place where cropping was applied should be marked (with a box), and molecular weight/size standards should be labeled wherever possible. For capillary electrophoresis assays, each trace in the graph should be color-coded and labeled to indicate which protein, gene, or sample is being measured (please try to avoid red/green combinations to accommodate our color-blind readers).

Source Data files will be made available to reviewers during evaluation of revised manuscripts and, if your paper is eventually published in JCB, the files will be directly linked to specific figures in the published article. Source Data Figures should be provided as individual PDF files (one file per figure). Authors should endeavor to retain a minimum resolution of 300 dpi or pixels per inch. Please review our instructions for export from Photoshop, Illustrator, and PowerPoint here: https://rupress.org/jcb/pages/submission-guidelines#revised

The typical timeframe for revisions is three to four months. If you anticipate any difficulties in meeting this aforementioned revision time limit, please contact us and we can work with you to find an appropriate time frame for resubmission. Please note that papers are generally considered through only one revision cycle, so any revised manuscript will likely be either accepted or rejected.

When submitting the revision, please include a cover letter addressing the reviewers' comments point by point. Please also highlight all changes in the text of the manuscript.

We hope that the comments below will prove constructive as your work progresses. We would be happy to discuss them further once you've had a chance to consider the points raised in this letter.

Thank you for this interesting contribution to Journal of Cell Biology. You can contact us at the journal office with any questions at cellbio@rockefeller.edu.

Sincerely,

Valerie Castellani, PhD
Monitoring Editor
Journal of Cell Biology

Dan Simon, PhD
Scientific Editor
Journal of Cell Biology

Reviewer #1 (Comments to the Authors (Required)):

A well written paper which identifies the chemokine Cxcl14 as a novel instructive cue for pioneer axon guidance, extension, turning and fasciculation in the zebrafish inner ear. The loss of this signaling was shown to perturb axonal navigation, and synaptic organization in the hair cells.
The conclusions drawn from the findings were overall well-supported, the time-lapse imaging is convincing though somewhat qualitative. however the manuscript would benefit from providing more information on what is known about the signaling distance of Cxcl14 and which cells express the chemokine within the inner ear. Moreover, what the potential role of Cxcl14 is in hair cell maturation and hair cell MET channel activity - is this a primary effect or secondary effect for example. Staining for synapses in hair cells will also better inform innervation. Finally, the reliance on the startle response as a readout of dysfunction in the mutants lacks a level of specificity as this can also be affected by cells of the lateral line and also motor neuron deficits.

minor
should remove this sentence from p4: This chemokine emerges as a potential candidate to mediate reinnervation of HCs after injury.
p.4 why stain only irx1a and not others? are there diff subpopulations of pll?
fig 2F- can you make this bigger?

Reviewer #2 (Comments to the Authors (Required)):

The manuscript by Rumbo and colleagues, submitted to JCB, is focused on the molecular mechanisms governing pioneer axons of the inner ear in zebrafish. The topic of pioneer axons, their behavior following contact with neuronal and non-neuronal cells

(guidepost cells and target area environment), received considerable attention over a 30-year span from the 1980s to early 2000s. Early efforts from the laboratory of David Bentley (UC Berkeley) provided a foundation for future molecular and genetic dissection of pioneer axon behavior in the laboratories of Corey Goodman, Haig Keshishian, Corey Bargmann, Mark Tessier-Lavigne (among many others). More recent work has revealed the influences of cell-cell interactions, morphogen gradients, as well as axonal overshoot and pruning. Among the most remarkable findings are those implicating positional information in the periphery and modality-specific targeting mechanisms in complex central neuropil.

The current paper under review resides in an area of research that is rich with history, phenomena, molecular mechanism and quantitative live imaging. In general, the new work is sound, relying primarily on descriptive live imaging as a tool to visualize pioneer axon behavior. The authors identify Cxcl14 and an important molecular player and nicely define how loss and ectopic expression influences pioneer axons. In many respects, this is classic developmental neuroscience, performed to a high standard. I do not have major concerns with the experiments as described and reported. But, I am surprised that this is considered at JCB. It seems more appropriate for a journal such as Development, or Genes and Development. The work is a nice addition to a large literature, but does not advance the field beyond the contribution of a new molecular mechanism and system-specific contributions of pioneer axons.

Reviewer #3 (Comments to the Authors (Required)):

Summary:
This manuscript investigates the cellular and molecular mechanisms underlying early assembly of the zebrafish vestibuloacoustic circuit. The authors identify a population of pioneer statoacoustic ganglion (SAG) neurons that share transcriptional features with anterior lateral line progenitors and demonstrate that these pioneer neurons establish an axonal scaffold required for both follower axon extension and coordinated neuroblast migration. Through a combination of high-resolution live imaging and CRISPR-mediated gene disruption, the authors identify the chemokine cxcl14 as a critical regulator of pioneer axon guidance, showing that loss of cxcl14 leads to reproducible pathfinding errors, reduced branching, and inappropriate target innervation. These anatomical findings are compelling, well supported by the data, and represent a meaningful contribution to our understanding of sensory circuit assembly.

The manuscript is well organized, clearly written, and the figures are of high quality. My primary concerns relate to the interpretation of the functional experiments. In my opinion, the current behavioral and functional analyses do not fully support the conclusion that the observed deficits arise specifically from impaired vestibuloacoustic function, as alternative explanations related to generalized locomotor or motor impairments have not been adequately excluded. In addition, the manuscript repeatedly states that cxcl14 is absent from lateral line hair cells, which appears to be inconsistent with published single-cell RNA-sequencing datasets and should be reconciled. Addressing these points would substantially strengthen the manuscript. Overall, I find the study to be novel, technically strong, and suitable for publication in the Journal of Cell Biology following satisfactory revision.

Major Concerns:
1) Behavioral consequences of crispants: My main concerns relate to the analysis and interpretation of the behavioral assays. The behavioral phenotype in the cxcl14 crispants is clear; however, it is less clear whether the observed deficits can be attributed specifically to impaired vestibuloacoustic function. The majority of crispant larvae exhibited significant reductions in spontaneous swimming activity and total distance traveled, while also displaying a reduced startle response to mechanical stimuli. Because these larvae are already markedly hypoactive, a diminished startle response alone does not provide conclusive evidence that the phenotype arises specifically from vestibuloacoustic miswiring. Indeed, if the orientation of the wells in Figure 9A' and B is fixed, it appears that the more active larvae are also those most likely to exhibit a startle response.

The behavioral analysis would also benefit from a more detailed characterization of the evoked responses. As presented, the analysis relies primarily on distance traveled following stimulation, making it difficult to distinguish true acoustovestibular startle responses from more general post-stimulus locomotion. Quantifying kinematic features such as response probability, C-bend latency, peak angular velocity, or the proportion of stimuli that elicited a stereotypical C-start would substantially strengthen the interpretation of these data.
Although baseline subtraction accounts for differences in spontaneous locomotor activity immediately preceding the stimulus, it does not control for a reduced capacity to generate an escape response. For example, a larva with impaired postural stability or reduced motor output could still exhibit a diminished response amplitude after baseline correction despite detecting the stimulus normally. Consequently, the normalization procedure does not, by itself, establish that the observed behavioral deficit is sensory rather than motor in origin.
One possible way to strengthen this conclusion would be to include a behavioral assay that assesses motor responsiveness independently of mechanosensory input. For example, testing responses to a non-mechanical stimulus, such as a looming visual stimulus or dark flash, would help determine whether crispant larvae retain the ability to execute an escape response when activated through an alternative sensory modality. Such an experiment would substantially strengthen the conclusion that the reduced response to mechanical stimulation specifically reflects impaired vestibuloacoustic function rather than a generalized reduction in locomotor capability.

The authors' interpretation is certainly consistent with the compelling anatomical phenotype and the demonstrated axon guidance defects. Furthermore, the reported rolling behavior and impaired postural control are highly suggestive of vestibular dysfunction. However, this observation was described only qualitatively and was not presented or quantified, making it difficult to evaluate the extent to which they support the proposed interpretation. As a result, the mechanical startle assay is confounded by the substantial reduction in baseline locomotor activity, making it difficult to disentangle deficits in vestibuloacoustic processing from broader impairments in motor performance.

2) cxcl14 presence in lateral line hair cells: My second major concern relates to the authors' repeated assertion that cxcl14 is not expressed in lateral line hair cells. This statement is made multiple times throughout the manuscript (e.g., lines 180-181 and 267-268) but is not accompanied by supporting references. However, publicly available single-cell RNA-sequencing datasets of the zebrafish lateral line identify cxcl14 transcripts in both immature and mature lateral line hair cells (e.g., Lush et al., 2019; Baek et al., 2022). These published datasets appear to be inconsistent with the manuscript's repeated claim that lateral line hair cells lack cxcl14 expression.

The authors should therefore clarify this discrepancy. If the expression patterns reported in these datasets differ because of developmental stage, expression level, or methodological sensitivity, this should be discussed explicitly. Alternatively, if the authors have independent evidence demonstrating an absence of cxcl14 expression in lateral line hair cells at the stages examined here, this evidence should be presented.

This point is important because the assumption that lateral line hair cells do not express cxcl14 underlies several aspects of the authors' interpretation. For example, in Figure 6E the lateral line is effectively treated as a negative control for hair cell function. If cxcl14 is expressed in lateral line hair cells, these interpretations require additional justification. Likewise, the behavioral phenotypes presented in Figure 9 become more difficult to attribute exclusively to defects in vestibuloacoustic circuitry, as lateral line dysfunction could also contribute to altered responses to mechanical stimulation or altered spontaneous swimming.

Finally, I noticed that Figure 3G appears to contain small puncta of expression surrounding the eye and posterior to the otic vesicle that may correspond to lateral line neuromasts, although this is difficult to determine from the current figure. If so, these observations would further support the need to clarify the expression pattern of cxcl14 in the lateral line.

Rather than representing a complication, expression of cxcl14 within the lateral line could broaden the impact of the study by suggesting that chemokine-mediated guidance mechanisms are shared across multiple mechanosensory systems. Clarifying this point would therefore strengthen both the rigor and the broader significance of the manuscript.

Minor Concerns:
Line 117-121: Please cite the sources for the previously defined markers to identify aLL and pLL neurons. Or, are the examples (alcama & hoxb5b) new findings from the sub-clustering?
Line 126: Citation required for the pLL pioneer marker gfra1a - Woodruff et al 2025?
Line 148: "The pioneer SAG axon-pLL axon interaction finally resolved" appears to be an incomplete sentence
Line 155: Figure 2F - control 35 hpf dashed line appears to be offset
Line 156-157: Quantification of the changes in posterior lobe could potentially be improved by considering not just the length and width but potentially the fluorescence intensity.
Line 187-189: It is unclear to me in the image provided how the two spots indicated by the arrows were identified as the hindbrain entry points.
Line 198: "complex" is suggested to be removed
Line 251-252: Can the relatively proximity of the cxcl14 source and the start of the rerouting be quantified?
Line 313: Citation required from Mauthner cell literature
Line 365-366: It is unclear how the expression pattern in HCs provides cues for function.
Line 427: A note regarding your hypothesis, it seems cxcl14 expression occurs shortly after lateral line hair cell ablation (0.5-1hr post-ablation), while the first hair cells emerge around 5-10 hrs post-ablation (Baek et al 2022).
Line 442-443: A section of text from the Photoconversion section appears to have been left behind.
Line 492: Within this section please be clear as to whether you were using larvae or embryos. Had the larvae emerged from the larvae? If not, were they manually dechorionated?
Line 587: Replace "1 image every 0.04 seconds" with 25 fps.
Line 792-795: Delete text about photoconversion.
Line 797: Figure 1C, please remove the small numbers on the y-axis.

September 2026

Response to reviewers

Rumbo, Bañón, et al., JCB manuscript #202604185

We thank the reviewers for the comments in our manuscript. We have followed their advice and added new data to answer their concerns. We provide a new version of the manuscript with amended text and figures that we hope will be suitable for publication in JCB.

Below we answer point-by-point the reviewers concerns:

Reviewer #1

A well written paper which identifies the chemokine Cxcl14 as a novel instructive cue for pioneer axon guidance, extension, turning and fasciculation in the zebrafish inner ear. The loss of this signaling was shown to perturb axonal navigation, and synaptic organization in the hair cells.
The conclusions drawn from the findings were overall well-supported, the time-lapse imaging is convincing though somewhat qualitative.

1. However the manuscript would benefit from providing more information on what is known about the signaling distance of Cxcl14

To our knowledge, there appears to be no papers directly quantifying or describing signalling distance / effective diffusion for Cxcl14 in zebrafish or other models. CXCL14 chemokine is highly conserved at the sequence level with CXCL12, its closest related CXC protein. For CXCL12, the effective tissue diffusion was mathematically modelled to be small (around 5-25μm^2/s); thus, it is plausible that Cxcl14 has a relatively short-distance signaling range as well (1). Moreover, previous studies also reported an autocrine signaling mode in tissue grafts (2).

(1) Venkiteswaran, G., Lewellis, S. W., Wang, J., Reynolds, E., Nicholson, C., & Knaut, H. (2013). Generation and dynamics of an endogenous, self-generated signaling gradient across a migrating tissue. Cell, 155(3), 674-687.
(2) Augsten, Martin, et al. "CXCL14 is an autocrine growth factor for fibroblasts and acts as a multi-modal stimulator of prostate tumor growth." *Proceedings of the National Academy of Sciences* 106.9 (2009): 3414-3419.

This has been added to Discussion, line 462.

2. Which cells express the chemokine within the inner ear.

We thank the reviewer for their question. To respond, we have performed HCR ISH with the HC marker cabp1b that presumptively labels extrastriolar HC at 36, 72 hpf and 5 dpf (1, 2). This has provided the following new information.

At 36 hpf, HCR shows that cxcl14 and cabp1b are co-expressed in a few Brn3c+ HC in the anterior macula and only in two HC cells in the posterior macula. In both maculae, other Brn3c+ HC are present but do not express cabp1b nor cxcl14. This suggests that cxcl14 is expressed in a subset of HC that corresponds to future extrastriolar HC. At 24 hpf, cxcl14 in the posterior region is expressed in the two tether cells. Thus, it is tempting to speculate that these two tether cells are the ones that retain cxcl14 expression at 36 hpf.

Also, by HCR cxcl14 labelling at 36 hpf, we find that it is expressed in future cristae domains. At this stage, Brn3c+ HC are not yet detected but we observed that few axons already project to these domains. This data further reinforces the idea that cxcl14 attracts axons to the new sites of HC emergence.

At 72 hpf, cxcl14 expression in both maculae disappears; instead, it is highly expressed in cristae, in cabp1b+/Brn3c+ HCs while it is absent in cabp1b-/ Brn3c+ HCs. Moreover, it is also detected in surrounding cells, which are not Brn3c+. Thus, these that could be supporting cells or differentiating HC precursors.

At 5 dpf, cxcl14 is maintained in cristae only in cells surrounding HCs.

(1) Shi, T., Beaulieu, M. O., Saunders, L. M., Fabian, P., Trapnell, C., Segil, N., Crump, J. G., & Raible, D. W. (2023). Single-cell transcriptomic profiling of the zebrafish inner ear reveals molecularly distinct hair cell and supporting cell subtypes. eLife, 12. https://doi.org/10.7554/eLife.82978

(2) Baeza-Loya, S., Bùi, J. T., & Raible, D. W. (2026). Activity and retinoic acid drive hair cell spatial patterning in the zebrafish utricle. Development (Cambridge, England), 153(6). https://doi.org/10.1242/DEV.205363/371091

We have included this information in Figure 3 (new panels F-G' and I-J''), results section (line 196-207) and in the discussion (line 417-419).

We would like to mention that we also purchased the probe for cabp2b (that labels striolar HCs) but it did not work after a few attempts.

3. Moreover, what the potential role of Cxcl14 is in hair cell maturation and hair cell MET channel activity - is this a primary effect or secondary effect for example. Staining for synapses in hair cells will also better inform innervation.

We agree with the reviewer that this is still an open question. As it has been published that the chemokine can have a paracrine and an autocrine roles, and we find effects on neurons but also in HCs, both signaling events could be taking place. As the receptor is unknown, it is difficult to assess if the effect on HCs is autocrine (direct effect) or due to loss of axonal targeting (indirect effect).

We also added the line 461-462 for clarity: The lack of a known receptor for Cxcl14 also impedes the study of the possible autocrine effect in HCs.

We also agree with the reviewer that visualizing synapses would add significant information about innervation. Unfortunately, we have tried to immunolabel CtBP and MAGUK (pre- and post-synaptic markers) using three different protocols and none of them have worked in our hands. The antibodies were specifically obtained to address this revision.

The whole mount protocols we followed are from Jukic, et al., 2024; Sheets, et al., 2017; and we also performed the immunostaining in cryosections.

Here we include two example images showing that we obtained a lot of background staining, being unable to distinguish what was background and what was real signal.

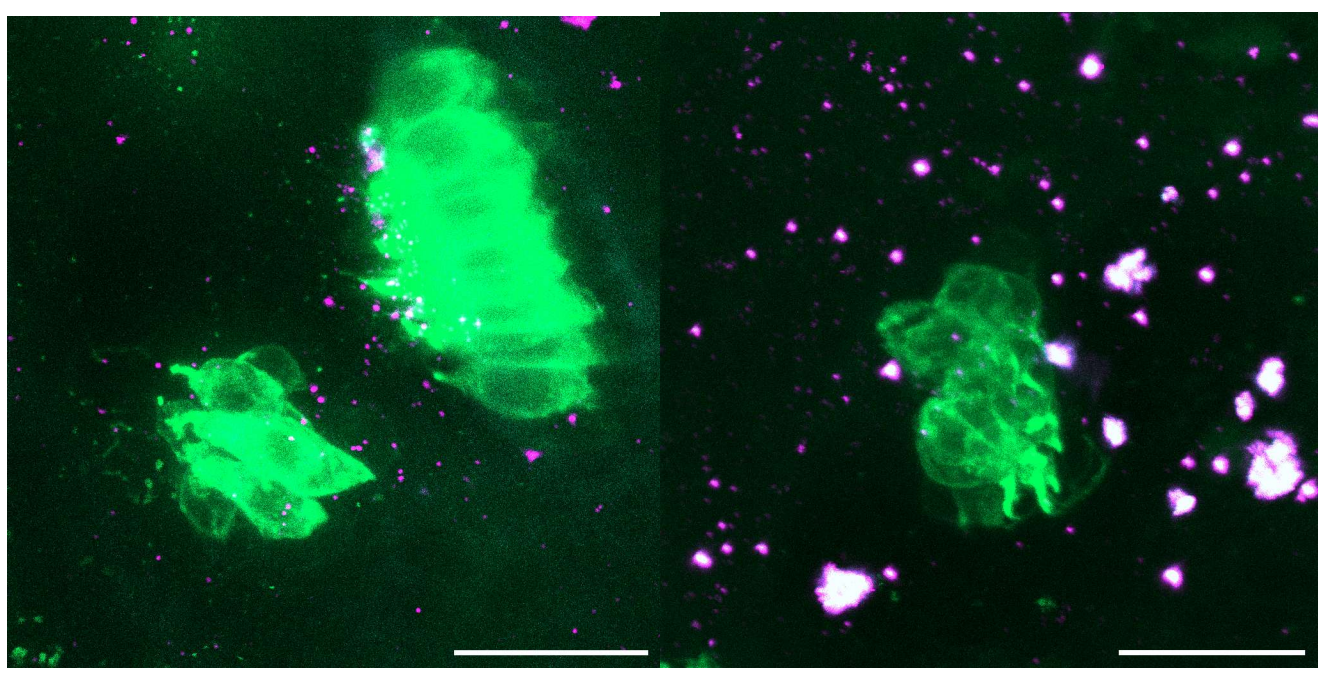

HCs labelled in green by Tg(Brn3c:GFP). Immunolabeling of CtBP (magenta) and MAGUK (red).

Therefore at this time, we cannot better inform if HC synapses are affected in embryos lacking Cxcl14.

4. Finally, the reliance on the startle response as a readout of dysfunction in the mutants lacks a level of specificity as this can also be affected by cells of the lateral line and also motor neuron deficits.

We appreciate this comment and agree that with the results presented in the original manuscript, we could not exclude motor neuron deficits nor that the lateral line was affected. In this new version, we have calculated the probability of response per tap instead of just showing the overall distance travelled after tapping stimuli (new panel Fig 8B'").
It is true that crispant larvae show a reduced general activity compared to control larvae, which might be the parameter indicating motor neuron deficits. Thus, we tested the locomotor capacity by adding a non-mechanosensory stimulus to check the reactivity of both groups. We chose a visual stimulus, specifically a light-to-dark sudden transition. If there was a locomotor impediment, crispant larvae should not be able to respond to this stimulus to the same extent. However, we observed that crispant larvae responded with no significant differences to the darkness, similar to control counterparts. In addition, the maximum velocity detected for all larvae (wild-type and crispants) did not display any significant differences either, indicating that crispants have the capacity to swim as fast as control larvae. These new results have been added in Figure 8 (new panels C-D'), results section (line 359-369). Furthermore, we added a detailed movie of the baseline conditions, showing that crispant larvae display less motility and cannot maintain a dorsal-up position, a sign of imbalance due to affected vestibular system (Suppl. Video 2).

Cxcl14 is not expressed in HC of the lateral line (new HCR panel Fig 3I'' and J'') and there is proper innervation of neuromasts in cxcl14 mutants (see new Suppl. Fig. 3), which favors a major role of Cxcl14 in the inner ear. However, as we have not tested rheotaxis in our mutant larvae, we cannot fully exclude the possibility that lateral line cells might be also affected in cxcl14 crispants (included in Discussion, lines 472-478).

Minor

Should remove this sentence from p4: This chemokine emerges as a potential candidate to mediate reinnervation of HCs after injury.

This sentence has been removed.

p.4 why stain only irx1a and not others? are there diff subpopulations of pll?

We had previous indications from our lab that this marker could be staining SAG pioneers; thus, we tried this marker first. As requested by the reviewer, we have added an additional marker, greb1l (new panels in Figure 1 (G-G')).
Interestingly, we can now confirm using both markers identified in the analysis of scRNA-seq neurod1+ cells, that there are different populations at 18 hpf (Fig 1D and new panels Fig. 1G,G'). Irx1a is only expressed in a subset of the presumptive aLL progenitors, the ones closer to the inner ear that might be the true SAG pioneers. In contrast, greb1l is not only co-expressed with irx1a in the SAG pioneers, but also in a population more anterior that we believe corresponds to the aLL precursors. This observation is consistent with our scRNA-seq data: the dotplot (Fig 1D) shows that irx1a is expressed in fewer cells than greb1l.

Regarding the second question addressing if there are different subpopulations of pLL, we cited the work in Woodruff et al., 2025. In this work, they differentiated two distinct populations of pLL neurons, pioneers and followers, and this is mentioned in the manuscript.

fig 2F- can you make this bigger?
We made this panel bigger and enlarged the lettering.

Reviewer #2:

The manuscript by Rumbo and colleagues, submitted to JCB, is focused on the molecular mechanisms governing pioneer axons of the inner ear in zebrafish. The topic of pioneer axons, their behavior following contact with neuronal and non-neuronal cells (guidepost cells and target area environment), received considerable attention over a 30-year span from the 1980s to early 2000s. Early efforts from the laboratory of David Bentley (UC Berkeley) provided a foundation for future molecular and genetic dissection of pioneer axon behavior in the laboratories of Corey Goodman, Haig Keshishian, Corey Bargmann, Mark Tessier-Lavigne (among many others). More recent work has revealed the influences of cell-cell interactions, morphogen gradients, as well as axonal overshoot and pruning. Among the most remarkable findings are those implicating positional information in the periphery and modality-specific targeting mechanisms in complex central neuropil.

The current paper under review resides in an area of research that is rich with history, phenomena, molecular mechanism and quantitative live imaging. In general, the new work is sound, relying primarily on descriptive live imaging as a tool to visualize pioneer axon behavior. The authors identify Cxcl14 and an important molecular player and nicely define how loss and ectopic expression influences pioneer axons. In many respects, this is classic developmental neuroscience, performed to a high standard. I do not have major concerns with the experiments as described and reported. But, I am surprised that this is considered at JCB. It seems more appropriate for a journal such as Development, or Genes and Development. The work is a nice addition to a large literature, but does not advance the field beyond the contribution of a new molecular mechanism and system-specific contributions of pioneer axons.

We kindly appreciate the comments from reviewer 2 and we thank him/her for thinking our experiments and manuscript do not present any major concerns. We believe this manuscript aligns with JCB journal, as indicated by the editors and the other two reviewers.

Reviewer #3:

Summary:
This manuscript investigates the cellular and molecular mechanisms underlying early assembly of the zebrafish vestibuloacoustic circuit. The authors identify a population of pioneer statoacoustic ganglion (SAG) neurons that share transcriptional features with anterior lateral line progenitors and demonstrate that these pioneer neurons establish an axonal scaffold required for both follower axon extension and coordinated neuroblast migration. Through a combination of high-resolution live imaging and CRISPR-mediated gene disruption, the authors identify the chemokine cxcl14 as a critical regulator of pioneer axon guidance, showing that loss of cxcl14 leads to reproducible pathfinding errors, reduced branching, and inappropriate target innervation. These anatomical findings are compelling, well supported by the data, and represent a meaningful contribution to our understanding of sensory circuit assembly.

The manuscript is well organized, clearly written, and the figures are of high quality. My primary concerns relate to the interpretation of the functional experiments. In my opinion, the current behavioral and functional analyses do not fully support the conclusion that the observed deficits arise specifically from impaired vestibuloacoustic function, as alternative explanations related to generalized locomotor or motor impairments have not been adequately excluded. In addition, the manuscript repeatedly states that cxcl14 is absent from lateral line hair cells, which appears to be inconsistent with published single-cell RNA-sequencing datasets and should be reconciled. Addressing these points would substantially strengthen the manuscript. Overall, I find the study to be novel, technically strong, and suitable for publication in the Journal of Cell Biology following satisfactory revision.

Major Concerns:
1) Behavioral consequences of crispants: My main concerns relate to the analysis and interpretation of the behavioral assays. The behavioral phenotype in the cxcl14 crispants is

clear; however, it is less clear whether the observed deficits can be attributed specifically to impaired vestibuloacoustic function. The majority of crispant larvae exhibited significant reductions in spontaneous swimming activity and total distance traveled, while also displaying a reduced startle response to mechanical stimuli. Because these larvae are already markedly hypoactive, a diminished startle response alone does not provide conclusive evidence that the phenotype arises specifically from vestibuloacoustic miswiring. Indeed, if the orientation of the wells in Figure 9A' and B is fixed, it appears that the more active larvae are also those most likely to exhibit a startle response.

We kindly appreciate these comments and suggestions. First, we would like to clarify that the tracking and heatmap shown in figure 8A,A' represent the duration of the tapping experiment and does not represent the movement of larvae during the accommodation period.

The reviewer is correct that the larvae more active are also those that perform better in startle response. When analyzing the movies in baseline conditions, we can observe that most larvae that move poorly during the startle response, are tilted and have difficulties keeping a dorsal-up position (this movie is included as Suppl video 2). Probably this is why they are less active.

We have added the tracking of a 10-minute period in light conditions to show basal activity of both groups, together with a 10-minute period of darkness (new panel Fig. 8C). This shows that indeed crispant larvae are less active in light conditions.

The behavioral analysis would also benefit from a more detailed characterization of the evoked responses. As presented, the analysis relies primarily on distance traveled following stimulation, making it difficult to distinguish true acousticovestibular startle responses from more general post-stimulus locomotion. Quantifying kinematic features such as response probability, C-bend latency, peak angular velocity, or the proportion of stimuli that elicited a stereotypical C-start would substantially strengthen the interpretation of these data.

Although baseline subtraction accounts for differences in spontaneous locomotor activity immediately preceding the stimulus, it does not control for a reduced capacity to generate an escape response. For example, a larva with impaired postural stability or reduced motor output could still exhibit a diminished response amplitude after baseline correction despite detecting the stimulus normally. Consequently, the normalization procedure does not, by itself, establish that the observed behavioral deficit is sensory rather than motor in origin. One possible way to strengthen this conclusion would be to include a behavioral assay that assesses motor responsiveness independently of mechanosensory input. For example, testing responses to a non-mechanical stimulus, such as a looming visual stimulus or dark flash, would help determine whether crispant larvae retain the ability to execute an escape response when activated through an alternative sensory modality. Such an experiment would substantially strengthen the conclusion that the reduced response to mechanical stimulation specifically reflects impaired vestibuloacoustic function rather than a generalized reduction in locomotor capability.

Due to the technical limitations that our setup presents, we could only add the response probability and the peak velocity, as our camera can only record at a velocity of 25 frames per second. In order to analyze the C-bend latency or the specifics of C-start movements, we would need a much more rapid camera.

We did follow the reviewer’s suggestions and added a behavioral assay with a non-mechanical stimulus, specifically a light-to-dark sudden transition. We observed that the response of the larvae is stimulus-dependent, as there are no significant differences between control and crispant larvaes when the lights turn off in terms of distance moved nor velocity. However, there is a significant difference between both groups when the stimulus is mechanical (tapping) (new panels Fig 8C-C”).

Finally, we have also measured the maximum velocity of all larvae and observed that both groups, control and crispant larvae, have the capacity to reach a similar maximum velocity. Taking into account that crispant larvae can respond to a dark stimulus and their maximum velocity is similar to the one in controls, we concluded that the locomotor capacity is not affected (new panels Fig 8D-D’, new results added to lines 359-369).

The authors' interpretation is certainly consistent with the compelling anatomical phenotype and the demonstrated axon guidance defects. Furthermore, the reported rolling behavior and impaired postural control are highly suggestive of vestibular dysfunction. However, this observation was described only qualitatively and was not presented or quantified, making it difficult to evaluate the extent to which they support the proposed interpretation. As a result, the mechanical startle assay is confounded by the substantial reduction in baseline locomotor activity, making it difficult to disentangle deficits in vestibuloacoustic processing from broader impairments in motor performance.

We agree with the reviewer that although our interpretation of the behavior analysis might be consistent with the axon guidance defects, we must quantitatively report the number of larvae with stationary balance impairments and impaired postural control. We have added this to the text (line 370-375), together with a supplementary video (Suppl. video 2) showing basal activity of crispant and control larvae to show the differences in the movement of mutant larvae.

2) cxcl14 presence in lateral line hair cells: My second major concern relates to the authors' repeated assertion that cxcl14 is not expressed in lateral line hair cells. This statement is made multiple times throughout the manuscript (e.g., lines 180-181 and 267-268) but is not accompanied by supporting references. However, publicly available single-cell RNA-sequencing datasets of the zebrafish lateral line identify cxcl14 transcripts in both immature and mature lateral line hair cells (e.g., Lush et al., 2019; Baek et al., 2022). These published datasets appear to be inconsistent with the manuscript's repeated claim that lateral line hair cells lack cxcl14 expression.

The authors should therefore clarify this discrepancy. If the expression patterns reported in these datasets differ because of developmental stage, expression level, or methodological sensitivity, this should be discussed explicitly. Alternatively, if the authors have independent evidence demonstrating an absence of cxcl14 expression in lateral line hair cells at the stages examined here, this evidence should be presented.

This point is important because the assumption that lateral line hair cells do not express cxcl14 underlies several aspects of the authors' interpretation. For example, in Figure 6E the lateral line is effectively treated as a negative control for hair cell function. If cxcl14 is expressed in lateral line hair cells, these interpretations require additional justification.

Likewise, the behavioral phenotypes presented in Figure 9 become more difficult to attribute exclusively to defects in vestibuloacoustic circuitry, as lateral line dysfunction could also contribute to altered responses to mechanical stimulation or altered spontaneous swimming.

Finally, I noticed that Figure 3G appears to contain small puncta of expression surrounding the eye and posterior to the otic vesicle that may correspond to lateral line neuromasts, although this is difficult to determine from the current figure. If so, these observations would further support the need to clarify the expression pattern of cxcl14 in the lateral line.

Rather than representing a complication, expression of cxcl14 within the lateral line could broaden the impact of the study by suggesting that chemokine-mediated guidance mechanisms are shared across multiple mechanosensory systems. Clarifying this point would therefore strengthen both the rigor and the broader significance of the manuscript.

We did claim that Cxcl14 was not expressed in lateral line HC because we had performed several times ISH at 72hpf (3dpf) with different protocols to make sure we were not destroying lateral line HCs. We could never detect cxcl14 in HC in chromogenic or fluorescent ISH.

We were aware that scRNA-seq datasets detected cxcl14 expression in HC of the lateral line. Those datasets that the reviewer mentions were obtained from 5 dpf larvae. In our work, we only assessed the expression of cxcl14 until 3dpf; thus, due to developmental stage differences the expression levels could differ.

In the revised MS, we used HCR, a more sensitive technique, to detect the expression of cxcl14. We have been able to observe low expression of cxcl14 surrounding neuromast HCs by 3 dpf and at 5 dpf, suggesting cxcl14 presence in supporting or precursor cells. However, we have never detected it in Brn3c+ HCs as it happens in the early born cabp1b-expressing HC in the inner ear sensory patches. Thus, cxcl14 distribution in HCs versus support cells is different in the inner ear compared to neuromasts.

We have clarified this in the manuscript (line 208-210) and added the new images of cxcl14 expression surrounding HC neuromasts to figure 3 (panels I'' and J").

Minor Concerns:

Line 117-121: Please cite the sources for the previously defined markers to identify aLL and pLL neurons. Or, are the examples (alcama & hoxb5b) new findings from the sub-clustering?
Line 126: Citation required for the pLL pioneer marker gfra1a - Woodruff et al 2025?

We have added the corresponding citation for these two minor concerns (Woodruff, et al., 2025).

Line 148: "The pioneer SAG axon-pLL axon interaction finally resolved" appears to be an incomplete sentence

We have changed the sentence to: "The pioneer SAG axon-pLL axon interaction finally resolved by 33.5hpf.

Line 155: Figure 2F - control 35 hpf dashed line appears to be offset

We have fixed the dashed line.

Line 156-157: Quantification of the changes in posterior lobe could potentially be improved by considering not just the length and width but potentially the fluorescence intensity.

Whereas we appreciate this comment, we believe it is not an adequate readout for morphological changes of the SAG since fluorescence intensity presents a certain level of variability among embryos. Thus, we prefer to only display the length and width measurements.

Line 187-189: It is unclear to me in the image provided how the two spots indicated by the arrows were identified as the hindbrain entry points.

Parts of the text were changed for clarification and we also changed the reference from Fig. 3E to 3D,D'', as axons are more clearly visualized in those.

New paragraph: Analysis of cxcl14 expression combined with the visualization of axons by the TgBAC(neurod1:eGFP)^{nl1} axonal staining at 36 hpf, revealed that cxcl14 expression in these two spots corresponded with the entrance of central projections of SAG and LL to the hindbrain. We refer to these regions as entry points of SAG and LL central projections to the hindbrain (Fig. 3D,D''E, magenta and white arrowheads).

Line 198: "complex" is suggested to be removed

We removed "complex" from this sentence.

Line 251-252: Can the relatively proximity of the cxcl14 source and the start of the rerouting be quantified?

We quantified the distance between the ectopic cxcl14 source and the start of the rerouting as well as the length of the projection path of the rerouted axon to analyse their correlation-We also added the distance between the tip of the rerouted axon and the cxcl14 expressing cell (new panel Fig 5B).

Line 313: Citation required from Mauthner cell literature

Added the citations: Tabor, et al., 2014; Hela, et al., 2016.

Line 365-366: It is unclear how the expression pattern in HCs provides cues for function.

We modified the sentence to: The highly dynamic and specific expression pattern of cxcl14 in first-born HCs and then in specific HCs made us hypothesize that its function could be related to innervation.

Line 427: A note regarding your hypothesis, it seems cxcl14 expression occurs shortly after lateral line hair cell ablation (0.5-1hr post-ablation), while the first hair cells emerge around 5-10 hrs post-ablation (Baek et al 2022).

Indeed, it would be interesting to analyze the role of cxcl14 in regenerating HC. We are thinking of following this in the future. Lines 430-432 added in discussion.

Line 442-443: A section of text from the Photoconversion section appears to have been left behind.

This section was amended.

Line 492: Within this section please be clear as to whether you were using larvae or embryos. Had the larvae emerged from the larvae? If not, were they manually dechorionated?

To clarify this point, we modified the revised text as follows: Larvae were maintained as described until 72 hpf. Dechorionated larvae were, anesthetized using MS-222 (Tricaine) (42 μL of Tricaine (4mg/mL) per 1 mL of embryo medium)

Line 587: Replace "1 image every 0.04 seconds" with 25 fps.

We replaced this as the reviewer suggested.

Line 792-795: Delete text about photoconversion.

We deleted the text from this figure legend.

Line 797: Figure 1C, please remove the small numbers on the y-axis.

We removed the numbers as the reviewer suggested.

October 12, 2026

RE: JCB Manuscript #202604185R

Berta Alsina
Universitat Pompeu Fabra

Dear Dr. Alsina,

Thank you for submitting your revised manuscript entitled "The Cxcl14 chemokine defines pioneer axon guidance and early circuit assembly in the inner ear." We would be happy to publish your paper in JCB pending final revisions necessary to meet our formatting guidelines (see details below).

To avoid unnecessary delays in the acceptance and publication of your paper, please read the following information carefully.

A. MANUSCRIPT ORGANIZATION AND FORMATTING:

Full guidelines are available on our Instructions for Authors page, https://jcb.rupress.org/submission-guidelines#revised.
Submission of a paper that does not conform to JCB guidelines will delay the acceptance of your manuscript.

1) Text limits: Character count for Articles is < 40,000, not including spaces. Count includes title page, abstract, introduction, results, discussion, and acknowledgments. Count does not include materials and methods, figure legends, references, tables, or supplemental legends.

2) Figure formatting: Articles may have up to 10 main text figures. Scale bars must be present on all microscopy images, including inset magnifications. Please add scale bars to figures 3A-D,H and S2.

Also, please avoid pairing red and green for images and graphs to ensure legibility for color-blind readers. If red and green are paired for images, please ensure that the particular red and green hues used in micrographs are distinctive with any of the colorblind types. If not, please modify colors accordingly or provide separate images of the individual channels.

3) Statistical analysis: Error bars on graphic representations of numerical data must be clearly described in the figure legend. The number of independent data points (n) represented in a graph must be indicated in the legend. Please indicate whether 'n' refers to technical or biological replicates (i.e. number of analyzed cells, samples or animals, number of independent experiments). If independent experiments with multiple biological replicates have been performed, we recommend using distribution-reproducibility SuperPlots (please see Lord et al., JCB 2020) to better display the distribution of the entire dataset, and report statistics (such as means, error bars, and P values) that address the reproducibility of the findings.

Statistical methods should be explained in full in the materials and methods. For figures presenting pooled data the statistical measure should be defined in the figure legends. Please also be sure to indicate the statistical tests used in each of your experiments (both in the figure legend itself and in a separate methods section) as well as the parameters of the test (for example, if you ran a t-test, please indicate if it was one- or two-sided, etc.). Also, if you used parametric tests, please indicate if the data distribution was tested for normality (and if so, how). If not, you must state something to the effect that "Data distribution was assumed to be normal but this was not formally tested."

4) Materials and methods: Should be comprehensive and not simply reference a previous publication for details on how an experiment was performed. Please provide full descriptions (at least in brief) in the text for readers who may not have access to referenced manuscripts. The text should not refer to methods "...as previously described."

5) For all cell lines, vectors, strains, constructs/cDNAs, etc. - all genetic material: please include database / vendor ID (e.g. Addgene, ATCC, etc.) or if unavailable, please briefly describe their basic genetic features, even if described in other published work or gifted to you by other investigators (and provide references where appropriate). Please be sure to provide the sequences for all of your oligos: primers, si/shRNA, RNAi, gRNAs, etc. in the materials and methods. You must also indicate in the methods the source, species, and catalog numbers/vendor identifiers (where appropriate) for all of your antibodies, including secondary. If antibodies are not commercial, please add a reference citation if possible.

6) Microscope image acquisition: The following information must be provided about the acquisition and processing of images:
a. Make and model of microscope
b. Type, magnification, and numerical aperture of the objective lenses
c. Temperature
d. Imaging medium
e. Fluorochromes

f. Camera make and model
g. Acquisition software
h. Any software used for image processing subsequent to data acquisition. Please include details and types of operations involved (e.g., type of deconvolution, 3D reconstitutions, surface or volume rendering, gamma adjustments, etc.).

7) References: There is no limit to the number of references cited in a manuscript. References should be cited parenthetically in the text by author and year of publication. Abbreviate the names of journals according to PubMed.

8) Supplemental materials: Articles may have up to 5 supplemental figures and 10 videos. Please also note that tables, like figures, should be provided as individual, editable files. A summary of all supplemental material should appear at the end of the Materials and methods section. Please include one brief sentence per item.

9) Video legends: Should describe what is being shown, the cell type or tissue being viewed (including relevant cell treatments, concentration and duration, or transfection), the imaging method (e.g., time-lapse epifluorescence microscopy), what each color represents, how often frames were collected, the frames/second display rate, and the number of any figure that has related video stills or images.

10) eTOC summary: A ~40-50 word summary that describes the context and significance of the findings for a general readership should be included on the title page. The statement should be written in the present tense and refer to the work in the third person. It should begin with "First author name(s) et al..." to match our preferred style.

11) Conflict of interest statement: JCB requires inclusion of a statement in the acknowledgements regarding competing financial interests. If no competing financial interests exist, please include the following statement: "The authors declare no competing financial interests." If competing interests are declared, please follow your statement of these competing interests with the following statement: "The authors declare no further competing financial interests."

12) A separate author contribution section is required following the Acknowledgments in all research manuscripts. All authors should be mentioned and designated by their first and middle initials and full surnames. We encourage use of the CRediT nomenclature (https://casrai.org/credit/).

13) ORCID IDs: ORCID IDs are unique identifiers allowing researchers to create a record of their various scholarly contributions in a single place. Please note that ORCID IDs are required for all authors. At resubmission of your final files, please be sure to provide your ORCID ID and those of all co-authors.

14) Journal of Cell Biology now requires a data availability statement for all research article submissions. These statements will be published in the article directly above the Acknowledgments. The statement should address all data underlying the research presented in the manuscript. Please visit the JCB instructions for authors for guidelines and examples of statements at (https://rupress.org/jcb/pages/editorial-policies#data-availability-statement).

B. FINAL FILES:

Please upload the following materials to our online submission system. These items are required prior to acceptance. If you have any questions, contact JCB's Managing Editor, Lindsey Hollander (lhollander@rockefeller.edu).

-- An editable version of the final text (.DOC or .DOCX) is needed for copyediting (no PDFs).

-- High-resolution figure and MP4 video files: See our detailed guidelines for preparing your production-ready images, https://jcb.rupress.org/fig-vid-guidelines.

-- Cover images: If you have any striking images related to this story, we would be happy to consider them for inclusion on the journal cover. Submitted images may also be chosen for highlighting on the journal table of contents or JCB homepage carousel. Images should be uploaded as TIFF or EPS files and must be at least 300 dpi resolution.

It is JCB policy that if requested, original data images must be made available to the editors. Failure to provide original images upon request will result in unavoidable delays in publication. Please ensure that you have access to all original data images prior to final submission.

The license to publish form must be signed before your manuscript can be sent to production. A link to the license to publish form will be sent to the corresponding author only. Please take a moment to check your funder requirements before choosing the appropriate license.

Additionally, JCB encourages authors to submit a short video summary of their work. These videos are intended to convey the main messages of the study to a non-specialist, scientific audience. Think of them as an extended version of your abstract, or a short poster presentation. We encourage first authors to present the results to increase their visibility. The videos will be shared

on social media to promote your work. For more detailed guidelines and tips on preparing your video, please visit https://rupress.org/jcb/pages/submission-guidelines#videoSummaries.

Thank you for your attention to these final processing requirements. Please revise and format the manuscript and upload materials within 14 days. If you need an extension for whatever reason, please let us know and we can work with you to determine a suitable revision period.

Please contact the journal office with any questions at cellbio@rockefeller.edu.

Thank you for this interesting contribution, we look forward to publishing your paper in Journal of Cell Biology.

Sincerely,

Valerie Castellani, PhD
Monitoring Editor
Journal of Cell Biology

Dan Simon, PhD
Scientific Editor
Journal of Cell Biology
